# The effect of prior assumptions over the weights in BayesPI with application to study protein-DNA interactions from ChIP-based high-throughput data

**DOI:** 10.1186/1471-2105-11-412

**Published:** 2010-08-04

**Authors:** Junbai Wang

**Affiliations:** 1 Department of Pathology, The Norwegian Radium Hospital, Oslo University Hospital, Montebello 0310 Oslo, Norway

## Abstract

**Background:**

To further understand the implementation of hyperparameters re-estimation technique in Bayesian hierarchical model, we added two more prior assumptions over the weight in BayesPI, namely Laplace prior and Cauchy prior, by using the evidence approximation method. In addition, we divided hyperparameter (regularization constants α of the model) into multiple distinct classes based on either the structure of the neural networks or the property of the weights.

**Results:**

The newly implemented BayesPI was tested on both synthetic and real ChIP-based high-throughput datasets to identify the corresponding protein binding energy matrices. The results obtained were encouraging: 1) there was a minor effect on the quality of predictions when prior assumptions over the weights were altered (e.g. the prior probability distributions to the weights and the number of classes to the hyperparameters) in BayesPI; 2) however, there was a significant impact on the computational speed when tuning the weight prior in the model: for example, BayesPI with a Laplace weight prior achieved the best performance with regard to both the computational speed and the prediction accuracy.

**Conclusions:**

From this study, we learned that it is absolutely necessary to try different prior assumptions over the weights in Bayesian hierarchical model to design an efficient learning algorithm, though the quality of the final results may not be associated with such changes. In future, the evidence approximation method can be an alternative to Monte Carlo methods for computational implementation of Bayesian hierarchical model.

## Background

In our previous study, we developed a Bayesian neural network type of model - BayesPI - to study protein-DNA interactions, using ChIP-based high-throughput data [1]. In BayesPI, the model error function (data error) is interpreted as defining a likelihood function, and the model regularizer (a penalty term to the error function) corresponds to a prior probability distribution over the weights, and such a framework is considered as a Bayesian hierarchical model. In addition to the common model parameters, BayesPI includes unknown hyperparameters (e.g. weight decay rate *α *and model noise level *β*) that need to be learned from the data. There are three possible implementations to control the model hyperparameters when using Bayesian neural networks to infer the model parameters: 1) using Markov chain Monte Carlo methods to simulate the probability distribution - MCMC [2]; 2) integrating out the model hyperparameters analytically before the application of Gaussian approximation of posterior distribution, and subsequently maximizing the true posterior over the model parameters - Maximum A Posterior Probability (MAP) [3]; and 3) integrating out the model parameters first, and then maximizing the resulting evidence over the hyperparameters - the Evidence Approximation [4]. Descriptions of the first two implementations can be found in the earlier papers [2,3], and in this study, we will focus only on the last approach (the evidence approximation) implemented in BayesPI.

Three motivations inspired us to pursue an investigation on the effect of prior assumptions over the weights (the evidence approximation) in Bayesian neural networks to study protein-DNA interactions from ChIP-based high-throughput data: 1) With regard to others' concern, before BayesPI paper was published, we received some criticisms about the treatment of hyperparameters in Bayesian neural networks. For example, do alternative definitions of hyperparameters according to the model parameters (e.g. divide the hyperparameters *α *into several classes based on either the structure of neural networks or the property of the model parameters) strongly influence the model inference? 2) With regard to our own interest, how significant will a different assignment of prior distribution (e.g. Gaussian prior, Laplace prior or Cauchy prior) to weights affect the outcome of Bayesian neural networks (e.g. prediction accuracy and computational time cost)? 3) With respect to a general survey of the application of Bayesian inferences in ChIP-based experiments, we searched PubMed using the keywords "Bayesian, chip" or "Bayesian, ChIP-chip," and then downloaded the search results that had been recorded before May 28, 2010. From this search, we obtained 33 papers that contained the above-mentioned keywords. Subsequently, we carried out a literature study of these 33 papers. To our surprise, only 14 of the 33 papers had applied Bayesian methods on issues related to motif discovery (e.g. DNA binding site identification) by using ChIP-based high-throughput data, and the remaining 19 papers had applied Bayesian methods in data integration, clustering and network reconstructions, etc. A detailed examination of the 14 papers relevant to protein-DNA interaction study reveal that BayesPI applied used evidence approximation to solve the posterior distribution in Bayesian inference, while the remaining 12 papers utilized the sampling methods (e.g. MCMC and Gibbs sampling) to simulate the posterior distribution of the Bayesian models (one paper cannot be determined because of lack of method description; detailed information of the 33 papers is available in [Additional file 1: Supplemental Data]). Though the present implementation (the evidence approximation) in BayesPI for handling hyperparameters has been rarely applied earlier, there are clear advantages of using it to solve the data mining problems [5]. Thus, by being motivated by the last finding along with the earlier two inspirations, we decided to carry out a follow-up study on the effect of prior assumption over the weights in BayesPI. Our study may pave the way for the future development of evidence approximation in Bayesian inferences as well as for the further application of the Bayesian methods in bioinformatics research.

## Results

### Performance comparisons from simulated ChIP-chip datasets

**Figure 1:**
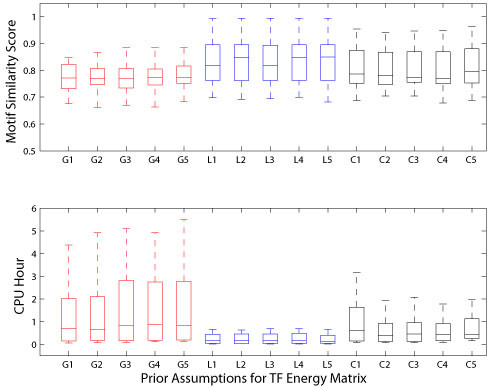
**Performance comparisons from simulated ChIP-chip datasets**. The upper panel of the figure shows the box plots of the distribution of motif similarity scores across 15 different weight prior configurations. The lower panel of the figures shows the box plots of the distribution of CPU hours used by 15 prior assumptions over the weights. Here, the red line represents Gaussian prior assumption to the weights (e.g. G1, G2, G3, G4, and G5), the blue line represents Laplace prior approximation over the weights (e.g. L1, L2, L3, L4, and L5), and the black line indicates Cauchy priors to the weights (C1, C2, C3, C4, and C5), in which the numerical values 1, 2, 3, 4, and 5 represent regularization constant α with one, two, three, four, and greater than five classes, respectively.

To evaluate the performance of BayesPI under (15) different prior assumptions over the weights, we first tried each of them on the same set of simulated ChIP-chip experiments (16 synthetic ChIP-chip datasets), where the synthetic DNA sequences and ChIP-chip log ratios were generated using MATLAB Bay Net toolbox and MATLAB build-in random number generator, respectively [1]. The accuracy of the predictions was accessed from motif similarity scores by comparing the predicted motif energy matrix with the corresponding SGD consensus sequences [6]. In Figure 1, we have illustrated the outcomes of the above-mentioned simulations in 15 different prior assumptions, where both the CPU hours required for the calculation and distribution of the motif similarity scores among all the tests are shown. The results are very interesting because no significant changes of the prediction quality could be observed across the tests after changing either the prior probability assumption or the number of subclasses for α hyperparameters, except for the tests with Gaussian approximation (e.g. comparing the distribution of motif similarity scores using Wilcoxon rank-sum test: Gaussian vs. Cauchy, p < 0.03; Laplace vs. Cauchy, p < 0.04). However, the CPU hours used for various tests differed significantly. Particularly, the selection of prior probability assumption over the weights in Bayesian neural networks had a much stronger impact on the cost of CPU hours than that by tuning the number of subclasses of hyperparameters. For examples, by using a Laplace assumption over the weights in BayesPI, the CPU hours used for the calculations were shortened by almost two to five times when compared with the assumptions of the weights by the other two probability distributions (e.g. comparing the distribution of used CPU hours by Wilcoxon rank-sum test: Gaussian vs. Laplace, p < 1.4e-9; Cauchy vs. Laplace, p < 5.8e-8). It is worth noting that the assignment of Laplace prior probability to weights utilizes the least CPU hours for the calculation, but provides the best prediction accuracy. Thus, we can expect Laplace approximation over the weights to provide the most efficient computation for BayesPI if real ChIP-chip datasets are used.

### Performance comparisons from real ChIP-chip datasets

**Figure 2:**
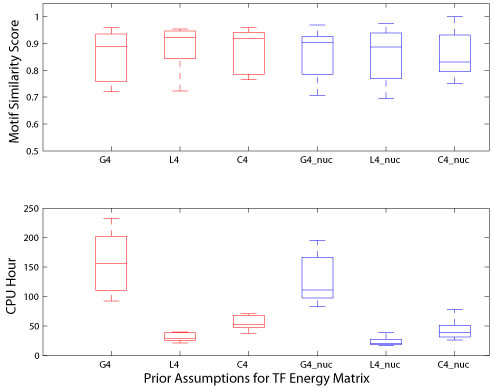
**Performance comparisons from real ChIP-chip datasets**. The upper panel of the figure shows the box plots of the distribution of motif similarity scores across six different weight prior configurations. The lower panel of the figure shows the box plots of the distribution of CPU hours used by six prior assumptions over the weights. Here, the red line represents the prior assumptions over the weights without inclusion of the nucleosome information, and the blue line indicates the prior assumptions over the weights with the inclusion of the nucleosome information. G4, L4, and C4 indicates Gaussian prior, Laplace prior, and Cauchy prior assumptions to the weights with four classes of regularization constants α, respectively.

After testing the effect of prior assumptions over the weights in BayesPI using the synthetic ChIP-chip datasets, we tried it on the real protein-DNA interaction datasets from ChIP-chip experiments. We collected ChIP-chip datasets for nine yeast TFs in rich medium condition [7], among which four (SWI4, INO4, ACE2, and XBP1) had the same consensus sequences as the TFs in the synthetic datasets. In the earlier tests, we neither found a significant variation in the prediction accuracy nor observed a strong perturbation of the computational time cost (Figure 1) through tuning the number of subclasses of α hyperparameters: hence, we decided to select only four subclasses for the hyperparameters in the rest of the studies. The results of these tests both with and without the inclusion of nucleosome information are presented in Figure 2 that shows that there is little difference in the prediction accuracies among the tests regarding the selection of prior probability assumptions and the inclusion of the nucleosome information. A comparison between the motif similarity scores provided by the three prior weight assumptions in BayesPI and those obtained by MatrixREDUCE is presented in Table 1. The results indicate that all poor predictions are caused by stress-induced transcription factors (e.g. ROX1, MSN2, and XBP1). Though BayesPI may provide some reasonable answers to TFs that are nonfunctional under certain growth conditions (e.g. Table 1, SKN7 and MSN2 in the YPD condition), its computational speed is much slower than that by the popular program MatrixREDUCE [8]. Nevertheless, the CPU hours used by BayesPI among the three prior weight assumptions differ significantly (e.g. comparing the distribution of used CPU hours without considering the nucleosome information by Wilcoxon rank-sum test: Gaussian vs. Laplace, p < 4.2e-5; Gaussian vs. Cauchy, p < 4.2e-5; Cauchy vs. Laplace, p < 2.9e-4). Furthermore, we found that the cost of the CPU hours for the computation was slightly reduced with regard to the nucleosome information. Taken together, it can be concluded that the computational efficiency of the Laplace prior assumption over the weights in the Bayesian neural networks clearly surpasses that of the other two weight priors, and that the Laplace prior may be suitable for the further improvement of BayesPI algorithm.

**Table 1:** Comparing motif similarity scores of nine yeast TFs from four different calculations.

TF Name (consensus sequence length)	Activated in stress conditions	BayesPI - Gaussian prior	BayesPI - Laplace prior	BayesPI - Cauchy prior	MatrixREDUCE
ACE2 (6)	No	0.89	0.95	0.96	0.90

**MSN2 (6)**	**Yes**[17]	**0.76**	0.93	**0.79**	**NA**

SWI4 (7)	No	0.96	0.94	0.94	0.95

YAP1 (7)	Yes[18]	0.93	0.92	0.92	0.93

INO4 (8)	No	0.90	0.92	0.94	0.97

**SKN7 (9)**	**Yes**[19]	0.86	0.87	0.86	**0.82**

FHL1 (10)	No	0.95	0.95	0.93	0.88

**ROX1 (12)**	**Yes**[20]	**0.72**	**0.72**	**0.78**	**0.75**

**XBP1 (12)**	**Yes**[21]	**0.76**	**0.77**	**0.76**	**NA**

For the nine yeast TFs, the ChIP-chip datasets were obtained from [7]; the regularization constants α in BayesPI were divided into four classes; MatrixREDUCE program was downloaded from the publication [8] and its default parameters were used in the present study. Here, the motif similarity scores greater than 0.85 represents a good match between the prediction and the SGD consensus sequences [1]. Poor predictions are marked by bold text. NA indicates that no results are available owing to the program reason. All the programs were applied on the same datasets and were run under a PC cluster (a dual-core CPU SUN X6220 blade node with 16 GB of RAM).

### Performance comparisons from human ChIP-Seq datasets

**Figure 3:**
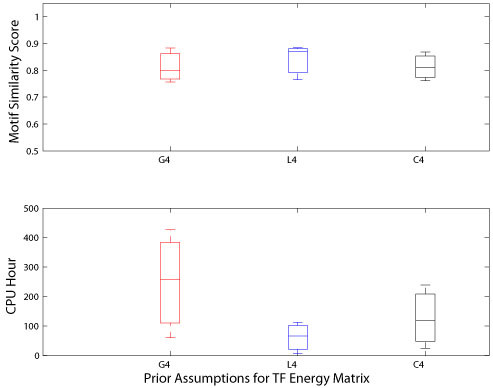
**Performance comparisons from human ChIP-Seq datasets**. The upper panel of the figure shows the box plots of the distribution of motif similarity scores across three different weight prior configurations. The lower panel of the figure shows the box plots of the distribution of CPU hours used by three prior assumptions over the weights. The red line represents Gaussian prior assumption over the weights, the blue line indicates the Laplace prior assumption over the weights, and the black line represents Cauchy prior assumption to the weights. Here, there are four distinct weight classes in the regularization constants α.

After the successful application of the earlier tests on ChIP-chip datasets, we tried the new BayesPI program on three human ChIP-Seq datasets [9] by applying three different prior assumptions (e.g. Gaussian, Lasso, and Cauchy) over the weights with predefined four groups of regularization constants α. Here, the inputs to BayesPI were pre-processed raw ChIP-Seq measurements, which are a set of putative protein binding sites (e.g. there are 5814, 26815, and 73957 putative TF binding sites for NRFS, CTCF, and STAT1, respectively.), and the corresponding tag densities obtained from SISSRs method [9]. The results of these tests are shown in Figure 3, which demonstrates that the Laplace prior requires much less CPU hours when compared with that required by the other two assumptions. For instance, to complete the same calculation, Laplace approximation needs only 50 percent to 25 percent of the CPU hours that is used by either Cauchy or Gaussian approximation. However, interestingly, the accuracy of the predictions does not differ significantly among various prior assumptions similar to the previous tests that employed the ChIP-chip datasets: tuning of the prior assumption over the weights does not seem to affect the quality of the predictions, but is rather observed to bring strong impact on the CPU requirement. Thus, a careful design of the weight priors in a Bayesian model may significantly reduce the computational cost for the calculation.

## Discussion

Nowadays, chromatin immunoprecipitation followed by massively paralleled sequencing (ChIP-Seq) is being used widely in various molecular biological researches such as investigating genome-wide protein-DNA interactions [7] and histone modification studies [10]. It is possible that the ChIP-Seq experiment may replace ChIP-chip technology completely [11] in future. That is because the ChIP-Seq experiment produces higher quality and higher resolution data than the ChIP-chip, which also avoids several pitfalls that accompany with the ChIP-chip technology: for example, array probe-specific behavior and dye bias [12]. In this work, we studied the effect of prior assumptions over the weight in BayesPI to predict the protein binding energy matrices from ChIP-based high-throughput datasets. The results on both synthetic and real experimental datasets were consistent: in general, the prior assumptions over the weights and the classification of regularization constants (e.g. hyperparameters *α*) into several classes did not strongly affect the final outcome of BayesPI (e.g. Figures 1, 2, and 3) if sufficient training datasets were provided; particularly, a change in the number of classes over the regularization constants had a much weaker impact on the requirement of computational resource than a change in the weight prior in BayesPI; nevertheless, the selection of prior approximation over the weights had the most significant influence on the CPU hours that were used for calculation (e.g. by using a Laplace prior, the computational time was reduced by more than 50 percent when compared with that utilized by the old BayesPI [1], a Gaussian prior.) Thus, the present study reveals the importance of defining a right weight prior to a Bayesian hierarchical model, which may dramatically speed up the calculation when the program is applied to a large dataset.

In addition to the above-mentioned findings that the computation efficiency of BayesPI is highly associated with prior assumptions over the weights, we also provided a detailed illustration of the hyperparameter re-estimation technique by using the evidence approximation method. We presume that the evidence method may become a popular approximate method for computational implementation of Bayesian hierarchical model (a deterministic algorithm), as well as become an alternative to Monte Carlo methods that are currently being widely used in bioinformatics research fields [13]. Particularly, the evidence method can overcome some of the inherent limitations of the sampling approaches, such as nonreproducible results, long burning period, and unknown stopping time.

## Conclusions

The present study has clarified several doubts in the early implementation of BayesPI: 1) prediction accuracy of BayesPI is robust against dividing the hyperparameters (e.g. regularization constants *α*) into multiple distinct groups; 2) there is a minor effect on the quality of predictions by selecting alternative prior assumptions over the weights in BayesPI; 3) however, there is a strong impact on the computational requirement for calculation when a proper weight prior is chosen. Overall, we have derived the new re-estimation formulas for both Laplace prior and Cauchy prior over the weights in the Bayesian neural networks, and the new implements have been tested successfully in both synthetic and real ChIP-based high-throughput datasets.

## Methods

### Computational modeling of protein-DNA interactions in BayesPI

In this study, we have only focused on the effect of prior assumption over the parameters in Bayesian neural networks. The descriptions of the biophysical background behind BayesPI and the implementation of the Bayesian predicative model to estimate the protein binding parameters by combining ChIP-based datasets with DNA sequence information will not be repeated, because they are available in the previous paper [1]. First, we regularized the objective function

(1) $$M(w)=\beta •E_{D}(D|w,\Lambda ,\eta )+\alpha •E_{w}(w|\Lambda ,\Gamma )$$

which can be used by the Bayesian neural networks [4] to determine the parameters (e.g. *w*, α, β). In the above-mentioned equation, *E*_*D*_, *E*_*w*_, *D*, and ⟨Λ, *η*, Γ⟩ are the model error function (data error), the model regularizer (a penalty term to the error function), the input data, and the hypothesis model space (e.g. Λ is the protein binding probability and Γ is the regularization function), respectively; *α *and *β *are the two unknown hyperparameters (e.g. weight decay rate and model noise level) that must be determined from the input data; and *w *indicates the model parameters (e.g. weights in the Bayesian neural networks), which represents the inferred the protein binding energy matrix and the chemical potentials from ChIP-based high-throughput data [1].

Here, a Gaussian model error function *E*_*D *_is assumed throughout as

(2) $$E_{D}=\frac{1}{2}\sum_{i=1}^{g}{\left(t_{i}-Y_{i}\right)}^{2}$$

where *t*_*i *_is the measured ChIP-based data to gene *i*, and *Y*_*i *_is the predicted TF occupancy data for that gene, according to a predefined TF binding probability (e.g. either inclusion or exclusion of nucleosome binding information in the protein-binding probability [1]). For the model regularizer *E*_*w*_, three types of weight prior assumptions were selected [3]: 1) Gaussian prior assumption over the weights,

(3) $$E_{w}=\frac{1}{2}\sum_{q=1}^{Q}w_{q}^{2}$$

in which *Q *is the number of parameters in the model, such as *w*; 2) Laplace prior distribution as the model regularization function,

(4) $$E_{w}=\sum_{q=1}^{Q}|w_{q}|$$

3) Cauchy prior assumption over the model parameters *w *in BayesPI

(5) $$E_{w}=\frac{1}{\alpha }\sum_{q=1}^{Q}\log(1+\alpha^{2}w_{q}^{2})$$

Based on the above-mentioned three weight priors, we applied the evidence approximation method [4] to determine the corresponding re-estimation formulas for both *α *and *β*, which can be used by Bayesian neural networks to fit the model (e.g. to learn the model parameters *w *from the data).

### Bayesian choice of α and β through the evidence approximation

#### Evidence approximation

Based on Bayes' theorem, a posterior distribution of the model parameters, can be given as

(6) $$P\left(w|D,\alpha ,\beta ,\Lambda ,\eta ,\Gamma \right)=\frac{\exp(-M(w))}{Z_{M}(\alpha ,\beta )}$$

where *M *is a probability framework of the objective function described in equation (1) and *Z*_*M *_is a normalization factor [4]. By employing a Gaussian approximation of the posterior probability, we have

(7) $$\begin{array}{l} P\left(w|D,\alpha ,\beta ,\Lambda ,\eta ,\Gamma \right)\\ \approx \frac{\exp(-M_{MP})\exp(-\frac{1}{2}{\left(w-w_{MP}\right)}^{T}A(w-w_{MP}))}{Z_{M}^{'}} \end{array}$$

in which $Z_{M}^{'}=\int dw\exp(-M_{MP}(w))$ and *A *is the Hessian of *M *(e.g. *A *= α∇∇*E*_*w *_+ β∇∇*E*_*D*_) evaluated at *w*_*MP*_. Here, we first assume that the most probable model parameters *w*_*MP *_are known (integrating out the model parameters), and then infer the hyperparameters through Bayes' rule

(8) $$\begin{array}{l} P\left(\alpha ,\beta |D,\Lambda ,\eta ,\Gamma \right)\\ =\frac{P(D|\alpha ,\beta ,\Lambda ,\eta ,\Gamma )P(\alpha ,\beta |\Lambda ,\eta ,\Gamma )}{P(D|\Lambda ,\Gamma ,\eta )} \end{array}$$

where we also assume equal priors P(α, β|Λ, *η*, Γ) to the alternative models and a constant term to the *P*(*D*|Λ, Γ, *η*). Thus

(9) P(α,β|D,Λ,η,Γ)≈P(D|α,β,Λ,η,Γ)

where P(*D*|α, β, Λ, *η*, Γ) is the evidence for the overall model,

(10) $$P\left(D|\alpha ,\beta ,\Lambda ,\eta ,\Gamma \right)=\frac{Z_{M}(\alpha ,\beta )}{Z_{w}(\alpha )Z_{D}(\beta )}$$

including both the model architecture and the regularizing parameters [4], where *Z*_*w*_(α) and *Z*_*D*_(β) are the normalization factors given by *Z*_*w*_(α) = ∫ *dw*exp(-αE_w_) and *Z*_*D*_(β) = ∫ *dD*exp(-βE_D_), respectively. By maximizing the log evidence of equation (10), we can determine the re-estimation formulas for hyperparameters α and β according to the weight assumptions *E*_*w *_in BayesPI.

#### Gaussian prior

The log evidence for hyperparameters is

(11) $$\log(P(D|\alpha ,\beta ,\Lambda ,\eta ,\Gamma ))=\log Z_{M}-\log Z_{w}-\log Z_{D}$$

where a Gaussian prior, equation (3), is used for *E*_*w *_and

(12) $$Z_{M}\approx \exp(-M_{MP}){\left(2\pi \right)}^{k/2}•\det A^{-1/2}$$

(13) $$Z_{w}\approx {\left(\frac{2\pi }{\alpha }\right)}^{k/2}$$

(14) $$Z_{D}\approx {\left(\frac{2\pi }{\beta }\right)}^{N/2}$$

After replacing *Z*_*M*_, *Z*_*w*_, *Z*_*D *_by equations (12), (13), and (14), respectively, equation (11) becomes

(15) $$\begin{array}{l} \log(P(D|\alpha ,\beta ,\Lambda ,\eta ,\Gamma ))\\ \approx -M_{MP}-\frac{1}{2}\log\det A+\frac{k}{2}\log\alpha -\frac{N}{2}\log2\pi +\frac{N}{2}\log\beta \end{array}$$

To determine the conditions that are satisfied at the maximum log evidence, we differentiated equation (15) with respect to *α *and *β*, and then set the derivative to zero from which we can obtain the re-estimation formulas for both *α *and *β *as follows

(16) $$\alpha =\frac{k-\alpha Trace(A^{-1}I)}{2E_{w}}$$

(17) $$\beta =\frac{N-k+\alpha Trace(A^{-1})}{2E_{D}}$$

Let

(18) $$\gamma =k-\alpha Trace(A^{-1})$$

The equations (16) and (17) can be rewritten as

(19) $$\alpha =\frac{\gamma }{2E_{w}}$$

(20) $$\beta =\frac{N-\gamma }{2E_{D}}$$

where *γ *are eigenvectors of *A*. For example, equation (18) can be transformed to

(21) $$\gamma =\sum_{q}\frac{\lambda_{q}}{\lambda_{q}+\alpha }$$

Where *λ*_*q *_are the eigenvalues of the β∇∇*E*_*D *_and the negative *λ*_*q *_are omitted from the sum. Thus, for a Gaussian weight prior, we used equation (21) to update the hyperparameters *α *and *β *through equations (19) and (20).

#### Laplace prior

By using equation (4) as a prior assumption over the weights, the Hessian of *M *becomes

(22) $$A=\beta \nabla \nabla E_{D}$$

The log evidence for the hyperparameters is

(23) $$\begin{array}{l} \log(P(D|\alpha ,\beta ,A,\eta ,R))\\ \approx -M_{MP}+\frac{k}{2}\log2\pi -\frac{1}{2}\log\det A-\log Z_{w}-\log Z_{D} \end{array}$$

where

(24) $$Z_{w}\approx {\left(\frac{2}{\alpha }\right)}^{k}$$

(25) $$Z_{D}\approx {\left(\frac{2\pi }{\beta }\right)}^{N/2}$$

After inserting equations (24) and (25) into the log evidence, we get

(26) $$\begin{array}{l} \log(P(D|\alpha ,\beta ,A,\eta ,R))\\ \approx -M_{MP}-\frac{1}{2}\log\det A+\frac{k}{2}\log2\pi -k\log2+k\log\alpha -\frac{N}{2}\log2\pi +\frac{N}{2}\log\beta \end{array}$$

To maximize the log evidence over the hyperparameters, we differentiated equation (26) with respect to *α *and *β*, and the derivative was set to zero, then we obtained the following re-estimation formulas

(27) $$\alpha =\frac{k}{E_{w}}$$

(28) $$\beta =\frac{N-k}{2E_{D}}$$

for the hyperparameters, when assuming a Laplace prior over the weights.

#### Cauchy prior

Here, we used equation (5) as the prior assumption over the weights, and the log evidence for the model can be given as

(29) $$\begin{array}{l} \log(P(D|\alpha ,\beta ,A,\eta ,R))\\ \approx -M_{MP}+\frac{k}{2}\log2\pi -\frac{1}{2}\log\det A-\log Z_{w}-\log Z_{D} \end{array}$$

where

(30) $$Z_{w}\approx {\left(\frac{\pi }{\left|\alpha \right|}\right)}^{k}$$

(31) $$Z_{D}\approx {\left(\frac{2\pi }{\beta }\right)}^{N/2}$$

After inserting equations (30) and (31) into equation (29), the log evidence becomes

(32) $$\begin{array}{c} L(\alpha ,\beta )\\ =-M_{MP}-\frac{1}{2}\log\det A+\frac{k}{2}\log2\pi -k\log\pi +k\log|\alpha |-\frac{N}{2}\log2\pi +\frac{N}{2}\log\beta \end{array}$$

where we assume that *α *is known for *E*_*w*_, *∇E*_*w*_, and *∇∇E*_*w*_. To determine the conditions suitable for the maximum log evidence, equation (32) was differentiated with respect to hyperparameters, and the derivative was set to zero. Then the re-estimation formulas for *α *and *β *are

(33) $$|\alpha |=\frac{k+\gamma }{2E_{w}}$$

(34) $$\beta =\frac{N-\gamma }{2E_{D}}$$

where

(35) $$\gamma =\sum_{q}\frac{\lambda_{q}}{\lambda_{q}+\alpha \nabla \nabla E_{w}}$$

where *λ*_*q *_are the eigenvalues of data error β∇∇*E*_*D*_. Thus, for a Cauchy prior, equation (35) can be used to compute hyperparameters α and β through equations (33) and (34). Detailed derivations of hyperparameters update functions for above three priors are available in [Additional file 1: Supplemental Methods].

#### Application of R-propagation algorithm

In equation (35), there is a second derivative ∇∇*E*_*w*_, which can be estimated from an efficient R-propagation algorithm of Pearlmutter [14]. The algorithm applies a differential operator R() on the Back-propagation neural networks. For example, let us assume that equation (5) is used by the model regularizer *E*_*w*_. Then

(36) $$E_{w}=\frac{1}{\alpha }a_{2}$$

where α_2 _is the node of the output layer

(37) $$a_{2}=\sum_{q}\log(1+H_{q}{}^{2})$$

in which *H*_*q *_is the node of the hidden layer

(38) $$H_{q}=\alpha a_{1}{}^{q}$$

and α_1_^*q *^is the node of the input layer

(39) $$a_{1}{}^{q}=w_{q}$$

After completing the above-mentioned forward computation of the neural networks, a backward pass can be subsequently obtained as

(40) $$\begin{array}{c} \frac{\partial E}{\partial a_{2}}=\frac{1}{\alpha }\\ \frac{\partial E}{\partial H_{q}}=\frac{1}{\alpha }\frac{2H_{q}}{1+H_{q}^{2}}\\ \frac{\partial E}{\partial a_{1}{}^{q}}=\frac{2H_{q}}{1+H_{q}^{2}}\\ \frac{\partial E}{\partial w_{q}}=\frac{2H_{q}}{1+H_{q}^{2}} \end{array}$$

and R-forward computation can be carried out as follows

(41) $$\begin{array}{lll} R(a_{1}{}^{q}) & = & V_{w_{q}}\\ R(H_{q}) & = & \alpha R(a_{1}{}^{q})\\ R(a_{2}) & = & \sum_{q}\frac{2H_{q}}{1+H_{q}^{2}}R(H_{q}) \end{array}$$

Furthermore, the R-backward computation can be carried out as follows

(42) $$R(\frac{\partial E}{\partial w_{q}})=\frac{-2(H_{q}^{2}-1)}{{\left(H_{q}^{2}+1\right)}^{2}}R(H_{q})$$

By following the above-mentioned R-back-propagation procedures, $R(\frac{\partial E}{\partial w_{q}})$ can be estimated, which is equivalent to computing the second derivative ∇∇*E*_*w *_[14]. Detailed description of application of R-propagation algorithm is available in [Additional file 1: Supplemental Methods]. The source code of BayesPI2 is public available http://folk.uio.no/junbaiw/bayesPI2.

### Multiple regularization constants α

For simplicity, we assumed that there is only one class of weights in BayesPI [1]. For example, the weights are modeled as coming from a single Gaussian prior (e.g. equation (3)). However, in a real study, weights may fall into multiple distinct groups [4]. Therefore, it is desirable to divide the weights into several classes *c*, with independent regularization constants α_*c*_. In the new version of BayesPI, there are five types of assignment of weight decay rate α to each of the three weight priors (e.g. Gaussian, Laplace, and Cauchy). The term αE_w _in equation (1) is replaced by $\sum_{c}\alpha_{c}E_{w}^{c}$, in which *c *is the number of classes to the regularization constants α: 1) if *c *equals 1, then all the weights have the same regularization constant α; 2) if *c *equals 2, then we can divide the weights into two groups, namely the weights in the hidden layer and the weights in the output layer; 3) if *c *equals 3, then it suggests that there are two distinct weight classes in the hidden layer (e.g. weights from the motif energy matrix and weight from the chemical potential), but only a single weight class in the output layer [1]); 4) if *c *equals 4, then it suggests that there are two independent weight classes in both the hidden layer and output layer; 5) if *c *is greater than 5, then it suggests that each binding position of the motif energy matrix has its own regularization constant α_c _as well as the chemical potential, and that the two weights in the output layer have their own regularization constants, respectively (e.g. if TF motif length equals 8, then the regularization constant *α *has 11 classes).

### Motif similarity score and Microarray datasets

To access the quality of the predicted motif binding sites, we used a published method (motif similarity score [15]) to estimate the similarity between the predicted motif energy matrices and the corresponding consensus sequences from the SGD database [16]. Detailed description of these calculations can be found in the previous publication [1]. Synthetic ChIP-chip datasets and real ChIP-chip experiments for nine yeast transcription factors were adopted from the earlier works [1,7]. ChIP-Seq datasets for three human TFs (STAT1, NRSF, and CTCF) were obtained from Jothi et al. [9]. More information about the preprocessing of both ChIP-chip and ChIP-Seq datasets are available in [1].

## Competing interests

The author declares that they have no competing interests.

## Authors' contributions

JW conceived and designed the study, implemented program, performed data analysis and drafted manuscript.

## Supplementary Material

Additional file 1 **Supplementary information to the paper**. Here we provide detailed description of derivation of hyperparameters update functions for three different priors (e.g. Gaussian, Cauchy, and Laplace), the implementation of R-propagation algorithm, and the full information of 33 papers that were obtained from PubMed on May 28^th^, 2010 by searching the keywords (e.g. Chip, Bayesian). Click here for file
