# Supplementary material for: The effect of prior assumptions over the weights in BayesPI with application to study protein-DNA interactions from ChIP-based high-throughput data
Source: BMC Bioinformatics. 2010 Aug 4;11:412. doi: 10.1186/1471-2105-11-412 (PMC2921412; doi:10.1186/1471-2105-11-412)
Supplement: Additional file 1 — Supplementary information to the paper. Here we provide detailed description of derivation of hyperparameters update functions for three different priors (e.g. Gaussian, Cauchy, and Laplace), the implementation of R-propagation algorithm, and the full information of 33 papers that were obtained from PubMed on May 28th, 2010 by searching the keywords (e.g. Chip, Bayesian). [file 1471-2105-11-412-S1.PDF]

Supplementary information to “The effect of prior assumptions over the weights in BayesPI with application to study protein-DNA interactions from ChIP-based high-throughput data”

Junbai Wang

Supplementary Methods

Derivation of hyperparameters update functions for Gaussian prior. The log evidence for hyperparameters is

$$\log(P(D \mid \alpha,\beta,\Lambda,\eta,\Gamma)) = \log Z_M - \log Z_w - \log Z_D \qquad [1]$$

where a Gaussian prior, equation $E_w = \frac{1}{2}\sum_{q=1}^{Q} w_q^2$, is used for E_w and

$$Z_M \approx \exp(-M_{MP})(2\pi)^{k/2} \bullet \det A^{-1/2} \qquad [2]$$

$$Z_w \approx \int_{-\infty}^{\infty} \exp(-\frac{1}{2}\alpha w^2) dw^k = (\frac{2\pi}{\alpha})^{k/2} \qquad [3]$$

$$Z_D \approx \int_{-\infty}^{\infty} \exp(-\frac{1}{2}\beta(y-t)^2) dt^N = (\frac{2\pi}{\beta})^{N/2} \qquad [4]$$

After replacing Z_M, Z_w, Z_D by equations [2], [3], and [4], respectively, equation [1] becomes

$$\log(P(D \mid \alpha,\beta,\Lambda,\eta,\Gamma)) \approx -M_{MP} + \frac{k}{2}\log 2\pi - \frac{1}{2}\log\det A - \frac{k}{2}2\pi + \frac{k}{2}\log\alpha - \frac{N}{2}\log 2\pi + \frac{N}{2}\log\beta \qquad [5]$$

Thus, the objective function is

$$L(\alpha,\beta) = -M_{MP} - \frac{1}{2}\log\det A + \frac{k}{2}\log\alpha - \frac{N}{2}\log 2\pi + \frac{N}{2}\log\beta \qquad [6]$$

To determine the conditions that are satisfied at the maximum log evidence, we differentiated equation [6] with respect to α and β, and then set the derivative to zero

$$\frac{\partial L}{\partial \alpha} = -E_w - \frac{1}{2}Trace(A^{-1}\frac{\partial A}{\partial \alpha}) + \frac{k}{2\alpha} = 0$$
$$\frac{\partial L}{\partial \beta} = -E_D - \frac{1}{2}Trace(A^{-1}\frac{\partial A}{\partial \beta}) + \frac{N}{2\beta} = 0 \qquad [7]$$

where

$$\frac{\partial A}{\partial \alpha} = \nabla\nabla E_w$$
$$\frac{\partial A}{\partial \beta} = \nabla\nabla E_D$$
$$\nabla\nabla E_w = I \qquad [8]$$
$$\nabla\nabla E_D = \frac{A - \alpha\nabla\nabla E_w}{\beta}$$

Based on equations [7] and [8], we can obtain the re-estimation formulas for both α and β as follows

$$\alpha = \frac{k - \alpha Trace(A^{-1}I)}{2E_w} \quad [9]$$

$$\beta = \frac{N - \beta Trace(A^{-1}\nabla\nabla E_D)}{2E_D}$$

$$= \frac{N - \beta Trace(A^{-1}\frac{A - \alpha\nabla\nabla E_w}{\beta})}{2E_D} \quad [10]$$

$$= \frac{N - Trace(I - A^{-1}\alpha\nabla\nabla E_w)}{2E_D}$$

$$= \frac{N - k + \alpha Trace(A^{-1})}{2E_D}$$

Let

$$\gamma = k - \alpha Trace(A^{-1}) \quad [11]$$

The equations [9] and [10] can be rewritten as

$$\alpha = \frac{\gamma}{2E_w} \quad [12]$$

$$\beta = \frac{N - \gamma}{2E_D} \quad [13]$$

where γ are eigenvectors of A. For example, we can diagonalize the total Hessian A

$$[V, D] = eig(A) \quad [14]$$

with the help of the following conditions

$$\begin{aligned} AV &= VD \\ A &= VDV^T \\ V^T V &= I \\ A^{-1} &= VD^{-1}V^T \\ I_g &= \nabla\nabla E_w \\ \gamma &= k - \alpha Trace(A^{-1}I_g) \end{aligned} \quad [15]$$

$$Trace(VD^{-1}V^T) = Trace(D^{-1}V^T V) = Trace(A^{-1})$$

Thus, equation [11] is transformed to

$$\begin{aligned} \gamma &= k - \alpha Trace(A^{-1}I_g) \\ &= k - \alpha Trace(D^{-1}V^T V I_g) \\ &= k - \alpha Trace(D^{-1}I_g) \\ &= k - \alpha Trace(\frac{I_g}{diag(\lambda) + \alpha I_g}) \\ &= \sum_q \frac{\lambda_q + \alpha I_g - \alpha I_g}{\lambda_q + \alpha} \\ &= \sum_q \frac{\lambda_q}{\lambda_q + \alpha} \end{aligned} \quad [16]$$

where $D = diag(\lambda) + \alpha I_g$, λ_q are the eigenvalues of the $\beta\nabla\nabla E_D$ and the negative λ_q are omitted from the sum. Thus, for a Gaussian weight prior, we used equation [16] to update the hyperparameters α and β through equations [12] and [13].

Derivation of hyperparameters update functions for Laplace prior. By using equation $E_w = \sum_{q=1}^{Q} | w_q |$ as a prior assumption over the weights, the Hessian of M becomes

$$\begin{aligned} A &= \alpha\nabla\nabla E_w + \beta\nabla\nabla E_D \\ &= \beta\nabla\nabla E_D \end{aligned} \qquad [17]$$

because $\nabla\nabla E_w = 0$. The log evidence for the hyperparameters is

$$\begin{aligned} &\log(P(D \mid \alpha, \beta, A, \eta, R)) \\ &\approx \log Z_M - \log Z_w - \log Z_D \qquad [18] \\ &\approx -M_{MP} + \frac{k}{2}\log 2\pi - \frac{1}{2}\log\det A - \log Z_w - \log Z_D \end{aligned}$$

where

$$Z_w \approx \int_{-\infty}^{\infty} \exp(-\alpha \mid w \mid) dw^k = (\frac{2}{\alpha})^k \qquad [19]$$

$$Z_D \approx \int_{-\infty}^{\infty} \exp(-\frac{1}{2}\beta(y-t)^2) dt^N = (\frac{2\pi}{\beta})^{N/2} \qquad [20]$$

After inserting equations [19] and [20] into the log evidence, we get

$$\begin{aligned} &\log(P(D \mid \alpha, \beta, A, \eta, R)) \\ &\approx -M_{MP} + \frac{k}{2}\log 2\pi - \frac{1}{2}\log\det A - k\log\frac{2}{\alpha} - \frac{N}{2}\log\frac{2\pi}{\beta} \end{aligned} \qquad [21]$$

Thus, the objective function is

$$\begin{aligned} &L(\alpha, \beta) \\ &= -M_{MP} - \frac{1}{2}\log\det A + \frac{k}{2}\log 2\pi - k\log 2 + k\log\alpha - \frac{N}{2}\log 2\pi + \frac{N}{2}\log\beta \end{aligned} \qquad [22]$$

To maximize the log evidence over the hyperparameters, we differentiated equation [22] with respect to α and β, and the derivative was set to zero

$$\frac{\partial L}{\partial \alpha} = -E_w - \frac{1}{2} Trace(A^{-1} \frac{\partial A}{\partial \alpha}) + \frac{k}{\alpha}$$
$$= -E_w - \frac{1}{2} Trace(A^{-1} \nabla\nabla E_w) + \frac{k}{\alpha}$$
$$= -E_w + \frac{k}{\alpha} = 0$$
$$\frac{\partial L}{\partial \beta} = -E_D - \frac{1}{2} Trace(A^{-1} \frac{\partial A}{\partial \beta}) + \frac{N}{2\beta} \qquad [23]$$
$$= -E_D - \frac{1}{2} Trace(A^{-1} \nabla\nabla E_D) + \frac{N}{2\beta}$$
$$= -E_D - \frac{1}{2} Trace(A^{-1} \frac{A - \alpha\nabla\nabla E_w}{\beta}) + \frac{N}{2\beta}$$
$$= -E_D - \frac{1}{2}\frac{k}{\beta} + \frac{N}{2\beta} = 0$$

Based on equation [23], we obtained the following re-estimation formulas

$$\alpha = \frac{k}{E_w} \qquad [24]$$
$$\beta = \frac{N-k}{2E_D} \qquad [25]$$

for the hyperparameters, when assuming a Laplace prior over the weights.

Derivation of hyperparameters update functions for Cauchy prior. Here, we used equation $E_w = \frac{1}{\alpha}\sum_{q=1}^{Q} \log(1+\alpha^2 w_q^2)$ as the prior assumption over the weights, and the log evidence for the model can be given as

$$\log(P(D \mid \alpha, \beta, A, \eta, R))$$
$$\approx \log Z_M - \log Z_w - \log Z_D \qquad [26]$$
$$\approx -M_{MP} + \frac{k}{2}\log 2\pi - \frac{1}{2}\log\det A - \log Z_w - \log Z_D$$

where

$$Z_w \approx \int_{-\infty}^{\infty} \exp(-\alpha \frac{1}{\alpha}\log(1+\alpha^2 w^2)) dw^k = (\frac{\pi}{|\alpha|})^k \qquad [27]$$

$$Z_D \approx \int_{-\infty}^{\infty} \exp(-\frac{1}{2}\beta(y-t)^2) dt^N = (\frac{2\pi}{\beta})^{N/2} \qquad [28]$$

After inserting equations [27] and [28] into equation [26], the log evidence becomes

$$L(\alpha, \beta)$$
$$= -M_{MP} - \frac{1}{2}\log\det A + \frac{k}{2}\log 2\pi - k\log\pi + k\log|\alpha| - \frac{N}{2}\log 2\pi + \frac{N}{2}\log\beta$$
[29]

where we assume that α is known for E_w, ∇E_w, and $\nabla\nabla E_w$. To determine the conditions suitable for the maximum log evidence, equation [29] was differentiated with respect to hyperparameters, and the derivative was set to zero

$$\begin{aligned}
\frac{\partial L}{\partial \alpha} &= -E_w - \frac{1}{2}Trace(A^{-1}\frac{\partial A}{\partial \alpha}) + \frac{k}{|\alpha|} \\
&= -E_w + \frac{k}{|\alpha|} - \frac{1}{2}Trace(A^{-1}\nabla\nabla E_w) = 0 \\
\frac{\partial L}{\partial \beta} &= -E_D - \frac{1}{2}Trace(A^{-1}\frac{\partial A}{\partial \beta}) + \frac{N}{2\beta} \\
&= -E_D + \frac{N}{2\beta} - \frac{1}{2}Trace(A^{-1}\nabla\nabla E_D) \\
&= -E_D - \frac{1}{2}Trace(A^{-1}\frac{A-\alpha\nabla\nabla E_w}{\beta}) + \frac{N}{2\beta} \\
&= -E_D - \frac{1}{2}\frac{k}{\beta} + \frac{1}{2\beta}Trace(A^{-1}\alpha\nabla\nabla E_w) + \frac{N}{2\beta} = 0
\end{aligned} \quad [30]$$

Thus,

$$\begin{aligned}
E_w &= \frac{k}{|\alpha|} - \frac{1}{2}Trace(A^{-1}\nabla\nabla E_w) \\
E_D &= -\frac{1}{2}\frac{k}{\beta} + \frac{1}{2\beta}Trace(A^{-1}\alpha\nabla\nabla E_w) + \frac{N}{2\beta}
\end{aligned} \quad [31]$$

and the re-estimation formulas for α and β are

$$|\alpha| = \frac{2k - |\alpha|\, Trace(A^{-1}\nabla\nabla E_w)}{2E_w} \quad [32]$$

$$\beta = \frac{-k + Trace(A^{-1}\alpha\nabla\nabla E_w) + N}{2E_D} \quad [33]$$

Let $\gamma = k - \alpha Trace(A^{-1}\nabla\nabla E_w)$ and $\alpha>0$, then equations [32] and [33] become

$$|\alpha| = \frac{k+\gamma}{2E_w} \quad [34]$$

$$\beta = \frac{N-\gamma}{2E_D} \quad [35]$$

Based on the conditions in equations [14] and [15], we obtain

$$\begin{aligned}
\gamma &= k - \alpha Trace(A^{-1}\nabla\nabla E_w) \\
&= k - \alpha Trace(\frac{1}{diag(\lambda) + \alpha\nabla\nabla E_w}\nabla\nabla E_w) \\
&= \sum_q \frac{\lambda_q + \alpha\nabla\nabla E_w - \alpha\nabla\nabla E_w}{\lambda_q + \alpha\nabla\nabla E_w} \\
&= \sum_q \frac{\lambda_q}{\lambda_q + \alpha\nabla\nabla E_w}
\end{aligned} \quad [36]$$

where λ_q are the eigenvalues of data error $\beta\nabla\nabla E_D$. Thus, for a Cauchy prior, equation [36] can be used to compute hyperparameters α and β through equations [34] and [35].

R-propagation algorithm. In equation [36], there is a second derivative $\nabla\nabla E_w$, which can be estimated from an efficient R-propagation algorithm of Pearlmutter [1]. The algorithm applies a differential operator R() on the Back-propagation neural networks. For example, let us assume that equation $E_w = \frac{1}{\alpha}\sum_{q=1}^{Q}\log(1+\alpha^2 w_q^2)$ is used by the model regularizer E_w. Then

$$E_w = \frac{1}{\alpha} a_2 \qquad [37]$$

where a_2 is the node of the output layer

$$\begin{aligned} a_2 &= \sum_q \log(1+\alpha^2 {w_q}^2) \\ &= \sum_q \log(1+{H_q}^2) \end{aligned} \qquad [38]$$

in which H_q is the node of the hidden layer

$$H_q = \alpha w_q = \alpha {a_1}^q \qquad [39]$$

and ${a_1}^q$ is the node of the input layer

$${a_1}^q = w_q \qquad [40]$$

After completing the above-mentioned forward computation of the neural networks, a backward pass can be subsequently obtained as

$$\begin{aligned} \frac{\partial E}{\partial a_2} &= \frac{1}{\alpha} \\ \frac{\partial E}{\partial H_q} &= \frac{\partial E}{\partial a_2}\frac{\partial a_2}{\partial H_q} = \frac{1}{\alpha}\frac{2H_q}{1+H_q^2} \\ \frac{\partial E}{\partial {a_1}^q} &= \frac{\partial E}{\partial a_2}\frac{\partial a_2}{\partial H_q}\frac{\partial H_q}{\partial {a_1}^q} = \frac{2H_q}{1+H_q^2} \\ \frac{\partial E}{\partial w_q} &= \frac{\partial E}{\partial a_2}\frac{\partial a_2}{\partial H_q}\frac{\partial H_q}{\partial {a_1}^q}\frac{\partial {a_1}^q}{\partial w_q} = \frac{2H_q}{1+H_q^2} \end{aligned} \qquad [41]$$

and R-forward computation can be carried out as follows

$$\begin{aligned} R({a_1}^q) &= V_{w_q} \\ R(H_q) &= R(\alpha {a_1}^q) = \alpha R({a_1}^q) \\ R(a_2) &= \sum_q \frac{\partial a_2}{\partial H_q} R(H_q) = \sum_q \frac{2H_q}{1+H_q^2} R(H_q) \end{aligned} \qquad [42]$$

Furthermore, the R-backward computation can be carried out as follows

$$
\begin{aligned}
R(\frac{\partial E}{\partial w_q}) &= R(\frac{2H_q}{1+H_q^2}) \\
&= \frac{\partial(\frac{2H_q}{1+H_q^2})}{\partial H_q} R(H_q) \qquad [43] \\
&= \frac{-2(H_q^2-1)}{(H_q^2+1)^2} R(H_q)
\end{aligned}
$$

By following the above-mentioned R-back-propagation procedures, $R(\frac{\partial E}{\partial w_q})$ can be estimated, which is equivalent to computing the second derivative $\nabla\nabla E_w$ [1].

Supplementary Data

PubMed Search Resulst

This message contains search results from the National Center for Biotechnology Information (NCBI) at the U.S. National Library of Medicine (NLM). Do not reply directly to this message

Sent on: Fri May 28 11:31:58 2010

Search: chip, bayesian

PubMed Results

Items 1 - 33 of 33

(Differential equation model) (Target gene detection)

1. Proc Natl Acad Sci U S A. 2010 Apr 27;107(17):7793-8. Epub 2010 Apr 12.

Model-based method for transcription factor target identification with limited data.

Honkela A, Girardot C, Gustafson EH, Liu YH, Furlong EE, Lawrence ND, Rattray M.

Department of Information and Computer Science, Aalto University School of Science and Technology, Helsinki, Finland. antti.honkela@tkk.fi

We present a computational method for identifying potential targets of a transcription factor (TF) using wild-type gene expression time series data. For each putative target gene we fit a simple differential equation model of transcriptional regulation, and the model likelihood serves as a score to rank targets. The expression profile of the TF is modeled as a sample from a Gaussian process prior distribution that is integrated out using a nonparametric Bayesian procedure. This results in a parsimonious model with relatively few parameters that can be applied to short time series datasets without noticeable overfitting. We assess our method using genome-wide chromatin immunoprecipitation (ChIP-chip) and loss-of-function mutant expression data for two TFs, Twist, and Mef2, controlling mesoderm development in Drosophila. Lists of top-ranked genes identified by our method are significantly enriched for genes close to bound regions identified in the ChIP-chip data and for genes that are differentially expressed in loss-of-function mutants. Targets of Twist display diverse expression profiles, and in this case a model-based approach performs significantly better than scoring based on correlation with TF expression. Our approach is found to be comparable or superior to ranking based on mutant differential expression scores. Also, we show how integrating complementary wild-type spatial expression data can further improve target ranking performance.

PMCID: PMC2867914 [Available on 2010/10/27]
PMID: 20385836 [PubMed - in process]

(Phylogenetic inference) (MCMC)
2. BMC Bioinformatics. 2010 Apr 12;11:184.

FPGA Acceleration of the phylogenetic likelihood function for Bayesian MCMC inference methods.

Zierke S, Bakos JD.

Department of Computer Science and Engineering, University of South Carolina, Columbia, SC, USA.

BACKGROUND: Likelihood (ML)-based phylogenetic inference has become a popular method for estimating the evolutionary relationships among species based on genomic sequence data. This method is used in applications such as RAxML, GARLI, MrBayes, PAML, and PAUP. The Phylogenetic Likelihood Function (PLF) is an important kernel computation for this method. The PLF consists of a loop with no conditional behavior or dependencies between iterations. As such it contains a high potential for exploiting parallelism using micro-architectural techniques. In this paper, we describe a technique for mapping the PLF and supporting logic onto a Field Programmable Gate Array (FPGA)-based co-processor. By leveraging the FPGA's on-chip DSP modules and the high-bandwidth local memory attached to the FPGA, the resultant co-processor can accelerate ML-based methods and outperform state-of-the-art multi-core processors. RESULTS: We use the MrBayes 3 tool as a framework for designing our co-processor. For large datasets, we estimate that our accelerated MrBayes, if run on a current-generation FPGA, achieves a 10x speedup relative to software running on a state-of-the-art server-class microprocessor. The FPGA-based implementation achieves its performance by deeply pipelining the likelihood computations, performing multiple floating-point operations in parallel, and through a natural log approximation that is chosen specifically to leverage a deeply pipelined custom architecture. CONCLUSIONS: Heterogeneous computing, which combines general-purpose processors with special-purpose co-processors such as FPGAs and GPUs, is a promising approach for high-performance phylogeny inference as shown by the growing body of literature in this field. FPGAs in particular are well-suited for this task because of their low power consumption as compared to many-core processors and Graphics Processor Units (GPUs).

PMCID: PMC2868009
PMID: 20385005 [PubMed - in process]

(Motif finding) (Gibbs sampling)
3. Biometrics. 2010 Jan 29. [Epub ahead of print]

Bayesian Modeling of ChIP-chip Data Through a High-Order Ising Model.

Mo Q, Liang F.

Department of Epidemiology and Biostatistics, Memorial Sloan-Kettering Cancer
Center, New York, New York 10065, U.S.A.

Summary. ChIP-chip experiments are procedures that combine chromatin immunoprecipitation (ChIP) and DNA microarray (chip) technology to study a variety of biological problems, including protein-DNA

interaction, histone modification, and DNA methylation. The most important feature of ChIP-chip data is that the intensity measurements of probes are spatially correlated because the DNA fragments are hybridized to neighboring probes in the experiments. We propose a simple, but powerful Bayesian hierarchical approach to ChIP-chip data through an Ising model with high-order interactions. The proposed method naturally takes into account the intrinsic spatial structure of the data and can be used to analyze data from multiple platforms with different genomic resolutions. The model parameters are estimated using the Gibbs sampler. The proposed method is illustrated using two publicly available data sets from Affymetrix and Agilent platforms, and compared with three alternative Bayesian methods, namely, Bayesian hierarchical model, hierarchical gamma mixture model, and Tilemap hidden Markov model. The numerical results indicate that the proposed method performs as well as the other three methods for the data from Affymetrix tiling arrays, but significantly outperforms the other three methods for the data from Agilent promoter arrays. In addition, we find that the proposed method has better operating characteristics in terms of sensitivities and false discovery rates under various scenarios.

PMID: 20128774 [PubMed - as supplied by publisher]

(Metropolis and Gibbs sampling) (Motif finding)

4. Bioinformatics. 2010 Mar 15;26(6):777-83. Epub 2010 Jan 28.

A hidden Ising model for ChIP-chip data analysis.

Mo Q, Liang F.

Department of Epidemiology and Biostatistics, Memorial Sloan-Kettering Cancer
Center, New York, NY 10065, USA. moq@mskcc.org

MOTIVATION: Chromatin immunoprecipitation (ChIP) coupled with tiling microarray (chip) experiments have been used in a wide range of biological studies such as identification of transcription factor binding sites and investigation of DNA methylation and histone modification. Hidden Markov models are widely used to model the spatial dependency of ChIP-chip data. However, parameter estimation for these models is typically either heuristic or suboptimal, leading to inconsistencies in their applications. To overcome this limitation and to develop an efficient software, we propose a hidden ferromagnetic Ising model for ChIP-chip data analysis. RESULTS: We have developed a simple, but powerful Bayesian hierarchical model for ChIP-chip data via a hidden Ising model. Metropolis within Gibbs sampling algorithm is used to simulate from the posterior distribution of the model parameters. The proposed model naturally incorporates the spatial dependency of the data, and can be used to analyze data with various genomic resolutions and sample sizes. We illustrate the method using three publicly available datasets and various simulated datasets, and compare it with three closely related methods, namely TileMap HMM, tileHMM and BAC. We find that our method performs as well as TileMap HMM and BAC for the high-resolution data from Affymetrix platform, but significantly outperforms the other three methods for the low-resolution data from Agilent platform. Compared with the BAC method which also involves MCMC simulations, our method is computationally much more efficient. AVAILABILITY: A software called iChip is freely available at http://www.bioconductor.org/. CONTACT: moq@mskcc.org.

PMID: 20110277 [PubMed - in process]

(Motif finding) (Stochastic sampling)

5. Nucleic Acids Res. 2010 Apr;38(7):2154-67. Epub 2010 Jan 6.

On the detection and refinement of transcription factor binding sites using ChIP-Seq data.

Hu M, Yu J, Taylor JM, Chinnaiyan AM, Qin ZS.

Center for Statistical Genetics, University of Michigan, Ann Arbor, Michigan
48109, USA.

Coupling chromatin immunoprecipitation (ChIP) with recently developed massively parallel sequencing technologies has enabled genome-wide detection of protein-DNA interactions with unprecedented sensitivity and specificity. This new technology, ChIP-Seq, presents opportunities for in-depth analysis of transcription regulation. In this study, we explore the value of using ChIP-Seq data to better detect and refine transcription factor binding sites (TFBS). We introduce a novel computational algorithm named Hybrid Motif Sampler (HMS), specifically designed for TFBS motif discovery in ChIP-Seq data. We propose a Bayesian model that incorporates sequencing depth information to aid motif identification. Our model also allows intra-motif dependency to describe more accurately the underlying motif pattern. Our algorithm combines stochastic sampling and deterministic 'greedy' search steps into a novel hybrid iterative scheme. This combination accelerates the computation process. Simulation studies demonstrate favorable performance of HMS compared to other existing methods. When applying HMS to real ChIP-Seq datasets, we find that (i) the accuracy of existing TFBS motif patterns can be significantly improved; and (ii) there is significant intra-motif dependency inside all the TFBS motifs we tested; modeling these dependencies further improves the accuracy of these TFBS motif patterns. These findings may offer new biological insights into the mechanisms of transcription factor regulation.

PMCID: PMC2853110
PMID: 20056654 [PubMed - indexed for MEDLINE]

(SNP)

6. BMC Proc. 2009 Dec 15;3 Suppl 7:S63.

Detecting single-nucleotide polymorphism by single-nucleotide polymorphism interactions in rheumatoid arthritis using a two-step approach with machine learning and a Bayesian threshold least absolute shrinkage and selection operator (LASSO) model.

González-Recio O, de Maturana EL, Vega AT, Engelman CD, Broman KW.

Department of Dairy Science, University of Wisconsin-Madison, 266 Animal Science
Building, 1675 Observatory Drive, Madison, Wisconsin 53706, USA.
ogrecio@gmail.com.

ABSTRACT : The objective of this study was to detect interactions between relevant single-nucleotide polymorphisms (SNPs) associated

with rheumatoid arthritis (RA). Data from Problem 1 of the Genetic Analysis Workshop 16 were used. These data consisted of 868 cases and 1,194 controls genotyped with the 500k Illumina chip. First, machine learning methods were applied for preselecting SNPs. One hundred SNPs outside the HLA region and 1,500 SNPs in the HLA region were preselected using information-gain theory. The software weka was used to reduce colinearity and redundancy in the HLA region, resulting in a subset of 6 SNPs out of 1,500. In a second step, a parametric approach to account for interactions between SNPs in the HLA region, as well as HLA-nonHLA interactions was conducted using a Bayesian threshold least absolute shrinkage and selection operator (LASSO) model incorporating 2,560 covariates. This approach detected some main and interaction effects for SNPs in genes that have previously been associated with RA (e.g., rs2395175, rs660895, rs10484560, and rs2476601). Further, some other SNPs detected in this study may be considered in candidate
gene studies.

PMCID: PMC2795964
PMID: 20018057 [PubMed - in process]

(Motif finding) (Evidence approximation)

7. BMC Bioinformatics. 2009 Oct 20;10:345.

BayesPI - a new model to study protein-DNA interactions: a case study of condition-specific protein binding parameters for Yeast transcription factors.

Wang J, Morigen.

Division of Pathology, The Norwegian Radium Hospital, Rikshospitalet University
Hospital, Montebello 0310 Oslo, Norway. junbai.wang@rr-research.no

BACKGROUND: We have incorporated Bayesian model regularization with biophysical modeling of protein-DNA interactions, and of genome-wide nucleosome positioning to study protein-DNA interactions, using a high-throughput dataset. The newly developed method (BayesPI) includes the estimation of a transcription factor (TF) binding energy matrices, the computation of binding affinity of a TF target site and the corresponding chemical potential. RESULTS: The method was successfully tested on synthetic ChIP-chip datasets, real yeast ChIP-chip experiments. Subsequently, it was used to estimate condition-specific and species-specific protein-DNA interaction for several yeast TFs. CONCLUSION: The results revealed that the modification of the protein binding parameters and the variation of the individual nucleotide affinity in either recognition or flanking sequences occurred under different stresses and in different species. The findings suggest that such modifications may be adaptive and play roles in the formation of the environment-specific binding patterns of yeast TFs and in the divergence of TF binding sites across the related yeast species.

PMCID: PMC2771022
PMID: 19857274 [PubMed - indexed for MEDLINE]

(Motif finding) (MCMC)

8. BMC Bioinformatics. 2009 Oct 26;10:352.

Bayesian modeling of ChIP-chip data using latent variables.

Wu M, Liang F, Tian Y.

Department of Statistics, Texas A&M University, College Station, TX 77843, USA.
mqwu@stat.tamu.edu

BACKGROUND: The ChIP-chip technology has been used in a wide range of biomedical studies, such as identification of human transcription factor binding sites,investigation of DNA methylation, and investigation of histone modifications in animals and plants. Various methods have been proposed in the literature for analyzing the ChIP-chip data, such as the sliding window methods, the hidden Markov model-based methods, and Bayesian methods. Although, due to the integrated consideration of uncertainty of the models and model parameters, Bayesian methods can potentially work better than the other two classes of methods, the existing Bayesian methods do not perform satisfactorily. They usually require multiple replicates or some extra experimental information to parametrize the model, and long CPU time due to involving of MCMC simulations. RESULTS: In this paper, we propose a Bayesian latent model for the ChIP-chip data. The new model mainly differs from the existing Bayesian models, such as the joint deconvolution model, the hierarchical gamma mixture model, and the Bayesian hierarchical model, in two respects. Firstly, it works on the difference between the averaged treatment and control samples. This enables the use of a simple model for the data, which avoids the probe-specific effect and the sample (control/treatment) effect. As a consequence, this enables an efficient MCMC simulation of the posterior distribution of the model, and also makes the model more robust to the outliers. Secondly, it models the neighboring dependence of probes by introducing a latent indicator vector. A truncated Poisson prior distribution is assumed for the latent indicator variable, with the rationale being justified at length. CONCLUSION: The Bayesian latent method is successfully applied to real and ten simulated datasets, with comparisons with some of the existing Bayesian methods, hidden Markov model methods, and sliding window methods. The numerical results indicate that the Bayesian latent method can outperform other methods, especially when the data contain outliers.

PMCID: PMC2779819
PMID: 19857265 [PubMed - indexed for MEDLINE]

(Motif finding) (MCMC)
9. BMC Bioinformatics. 2009 Sep 21;10:299.

BayesPeak: Bayesian analysis of ChIP-seq data.

Spyrou C, Stark R, Lynch AG, Tavaré S.

Statistical Laboratory, Centre for Mathematical Sciences, Wilberforce Road,
Cambridge, UK. C.Spyrou@statslab.cam.ac.uk

BACKGROUND: High-throughput sequencing technology has become popular and widely used to study protein and DNA interactions. Chromatin immunoprecipitation, followed by sequencing of the resulting samples, produces large amounts of data that can be used to map genomic

features such as transcription factor binding sites and histone modifications. METHODS: Our proposed statistical algorithm, BayesPeak, uses a fully Bayesian hidden Markov model to detect enriched locations in the genome. The structure accommodates the natural features of the Solexa/Illumina sequencing data and allows for overdispersion in the abundance of reads in different regions. Moreover, a control sample can be incorporated in the analysis to account for experimental and sequence biases. Markov chain Monte Carlo algorithms are applied to estimate the posterior distributions of the model parameters, and posterior probabilities are used to detect the sites of interest. CONCLUSION: We have presented a flexible approach for identifying peaks from ChIP-seq reads, suitable for use on both transcription factor binding and histone modification data. Our method estimates probabilities of enrichment that can be used in downstream analysis. The method is assessed using experimentally verified data and is shown to provide high-confidence calls with low false positive rates.

PMCID: PMC2760534
PMID: 19772557 [PubMed - indexed for MEDLINE]

(Classification)
10. Anal Chem. 2008 Dec 1;80(23):9005-12.

Identification of viruses using microfluidic protein profiling and Bayesian classification.

Fruetel JA, West JA, Debusschere BJ, Hukari K, Lane TW, Najm HN, Ortega J, Renzi
RF, Shokair I, VanderNoot VA.

Sandia National Laboratories, Livermore, California 94551-0969, USA. jfruet@sandia.gov

We present a rapid method for the identification of viruses using microfluidic chip gel electrophoresis (CGE) of high-copy number proteins to generate unique protein profiles. Viral proteins are solubilized by heating at 95 degrees C in borate buffer containing detergent (5 min), then labeled with fluorescamine dye (10 s), and analyzed using the microChemLab CGE system (5 min). Analyses of closely related T2 and T4 bacteriophage demonstrate sufficient assay sensitivity and peak resolution to distinguish the two phage. CGE analyses of four additional viruses--MS2 bacteriophage, Epstein-Barr, respiratory syncytial, and vaccinia viruses--demonstrate reproducible and visually distinct protein profiles. To evaluate the suitability of the method for unique identification of viruses, we employed a Bayesian classification approach. Using a subset of 126 replicate electropherograms of the six viruses and phage for training purposes, successful classification with non-training data was 66/69 or 95% with no false positives. The classification method is based on a single attribute (elution time), although other attributes such as peak width, peak amplitude, or peak shape could be incorporated and may improve performance further. The encouraging results suggest a rapid and simple way to identify viruses without requiring specialty reagents such as PCR probes and antibodies.

PMID: 19551975 [PubMed - indexed for MEDLINE]

(Motif finding) (MCMC)

11. Biometrics. 2009 Dec;65(4):1087-95.

A Bayesian hidden Markov model for motif discovery through joint modeling of genomic sequence and ChIP-chip data.

Gelfond JA, Gupta M, Ibrahim JG.

Department of Epidemiology and Biostatistics, Mail Code 7933, University of Texas
Health Science Center at San Antonio, 7703 Floyd Curl Drive, San Antonio, Texas
78229-3900, USA. gelfondjal@uthscsa.edu

We propose a unified framework for the analysis of chromatin (Ch) immunoprecipitation (IP) microarray (ChIP-chip) data for detecting transcription factor binding sites (TFBSs) or motifs. ChIP-chip assays are used to focus the genome-wide search for TFBSs by isolating a sample of DNA fragments with TFBSs and applying this sample to a microarray with probes corresponding to tiled segments across the genome. Present analytical methods use a two-step approach: (i) analyze array data to estimate IP-enrichment peaks then (ii) analyze the corresponding sequences independently of intensity information. The proposed model integrates peak finding and motif discovery through a unified Bayesian hidden Markov model (HMM) framework that accommodates the inherent uncertainty in both measurements. A Markov chain Monte Carlo algorithm is formulated for parameter estimation, adapting recursive techniques used for HMMs. In simulations and applications to a yeast RAP1 dataset, the proposed method has favorable TFBS discovery performance compared to currently available two-stage procedures in terms of both sensitivity and specificity.

PMCID: PMC2794970
PMID: 19210737 [PubMed - indexed for MEDLINE]

(Motif finding) (Sampling)
12. BMC Bioinformatics. 2008 Dec 12;9 Suppl 12:S7.

Extracting transcription factor binding sites from unaligned gene sequences with
statistical models.

Lu CC, Yuan WH, Chen TM.

Department of Electrical Engineering, National Tsing Hua University, Hsinchu
30013, Taiwan. cclu@ee.nthu.edu.tw

BACKGROUND: Transcription factor binding sites (TFBSs) are crucial in the regulation of gene transcription. Recently, chromatin immunoprecipitation followed by cDNA microarray hybridization (ChIP-chip array) has been used to identify potential regulatory sequences, but the procedure can only map the probable protein-DNA interaction loci within 1-2 kb resolution. To find out the exact binding motifs, it is necessary to build a computational method to examine the ChIP-chip array binding sequences and search for possible motifs representing the transcription factor binding sites. RESULTS: We developed a program to find out accurate motif sites from a set of unaligned DNA sequences in the yeast genome. Compared with MDscan, the prediction results suggest that, overall, our algorithm

outperforms MDscan since the predicted motifs are more consistent with previously known specificities reported in the literature and have better prediction ranks. Our program also outperforms the constraint-less Cosmo program, especially in the elimination of false positives. CONCLUSION: In this study, an improved sampling algorithm is proposed to incorporate the binomial probability model to build significant initial candidate motif sets. By investigating the statistical dependence between base positions in TFBSs, the method of dependency graphs and their expanded Bayesian networks is combined. The results show that our program satisfactorily extract transcription factor binding sites from unaligned gene sequences.

PMCID: PMC2638147
PMID: 19091030 [PubMed - indexed for MEDLINE]

(Classification)
13. Anal Chem. 2008 Nov 4. [Epub ahead of print]

Identification of Viruses Using Microfluidic Protein Profiling and Bayesian Classification.

Fruetel JA, West JA, Debusschere BJ, Hukari K, Lane TW, Najm HN, Ortega J, Renzi
RF, Shokair I, Vandernoot VA.

jfruet@sandia.gov.

We present a rapid method for the identification of viruses using microfluidic chip gel electrophoresis (CGE) of high-copy number proteins to generate unique protein profiles. Viral proteins are solubilized by heating at 95 degrees C in borate buffer containing detergent (5 min), then labeled with fluorescamine dye (10 s), and analyzed using the muChemLab CGE system (5 min). Analyses of closely related T2 and T4 bacteriophage demonstrate sufficient assay sensitivity and peak resolution to distinguish the two phage. CGE analyses of four additional viruses-MS2 bacteriophage, Epstein-Barr, respiratory syncytial, and vaccinia viruses-demonstrate reproducible and visually distinct protein profiles. To evaluate the suitability of the method for unique identification of viruses, we employed a Bayesian classification approach. Using a subset of 126 replicate electropherograms of the six viruses and phage for training purposes, successful classification with nontraining data was 66/69 or 95% with no false positives. The classification method is based on a single attribute (elution time), although other attributes such as peak width, peak amplitude, or peak shape could be incorporated and may improve performance further. The encouraging results suggest a rapid and simple way to identify viruses without requiring specialty reagents
such as PCR probes and antibodies.

PMID: 18980335 [PubMed - as supplied by publisher]

(DNA Methylation) (sampling)
14. Nat Biotechnol. 2008 Jul;26(7):779-85.

A Bayesian deconvolution strategy for immunoprecipitation-based DNA methylome analysis.

Down TA, Rakyan VK, Turner DJ, Flicek P, Li H, Kulesha E, Gräf S, Johnson N,
Herrero J, Tomazou EM, Thorne NP, Bäckdahl L, Herberth M, Howe KL, Jackson DK,
Miretti MM, Marioni JC, Birney E, Hubbard TJ, Durbin R, Tavaré S, Beck S.

Wellcome Trust Cancer Research UK Gurdon Institute, and Department of Genetics,
University of Cambridge, Tennis Court Road, Cambridge CB2 1QR, UK.
thomas.down@gurdon.cam.ac.uk

DNA methylation is an indispensible epigenetic modification required for regulating the expression of mammalian genomes. Immunoprecipitation-based methods for DNA methylome analysis are rapidly shifting the bottleneck in this field from data generation to data analysis, necessitating the development of better analytical tools. In particular, an inability to estimate absolute methylation levels remains a major analytical difficulty associated with immunoprecipitation-based DNA methylation profiling. To address this issue, we developed a cross-platform algorithm-Bayesian tool for methylation analysis (Batman)-for analyzing methylated DNA immunoprecipitation (MeDIP) profiles generated using oligonucleotide arrays (MeDIP-chip) or next-generation sequencing (MeDIP-seq). We developed the latter approach to provide a high-resolution whole-genome DNA methylation profile (DNA methylome) of a mammalian genome. Strong correlation of our data, obtained using mature human spermatozoa, with those obtained using bisulfite sequencing suggest that combining MeDIP-seq or MeDIP-chip with Batman provides a robust, quantitative and cost-effective functional genomic strategy for elucidating the function of DNA methylation.

PMCID: PMC2644410
PMID: 18612301 [PubMed - indexed for MEDLINE]

(networks) (Monte Carlo sampling)

15. EURASIP J Bioinform Syst Biol. 2008:248747.

Recovering genetic regulatory networks from chromatin immunoprecipitation and steady-state microarray data.

Zhao W, Serpedin E, Dougherty ER.

Electrical and Computer Engineering Department, Texas A&M University, College
Station, TX 77843, USA.

Recent advances in high-throughput DNA microarrays and chromatin immunoprecipitation (ChIP) assays have enabled the learning of the structure and functionality of genetic regulatory networks. In light of these heterogeneous data sets, this paper proposes a novel approach for reconstruction of genetic regulatory networks based on the posterior probabilities of gene regulations. Built within the framework of Bayesian statistics and computational Monte Carlo techniques, the proposed approach prevents the dichotomy of classifying gene interactions as either being connected or disconnected, thereby it reduces significantly the inference errors. Simulation results corroborate the superior performance of the proposed approach relative to the existing state-of-the-art algorithms. A genetic regulatory network for Saccharomyces cerevisiae

is inferred based on the published real data sets, and biological meaningful results are discussed.

PMCID: PMC2435223
PMID: 18584039 [PubMed - in process]

(Networks)
16. Genome Res. 2008 Aug;18(8):1314-24. Epub 2008 Jun 18.

Inferring causal relationships among different histone modifications and gene expression.

Yu H, Zhu S, Zhou B, Xue H, Han JD.

Chinese Academy of Sciences Key Laboratory of Molecular Developmental Biology,
Center for Molecular Systems Biology, Institute of Genetics and Developmental
Biology, Chinese Academy of Sciences, Beijing, China.

Erratum in:
Genome Res. 2008 Sep;18(9):1544.

Histone modifications are major epigenetic factors regulating gene expression. They play important roles in maintaining stem cell pluripotency and in cancer pathogenesis. Different modifications may combine to form complex "histone codes." Recent high-throughput technologies, such as "ChIP-chip" and "ChIP-seq," have generated high-resolution maps for many histone modifications on the human genome. Here we use these maps to build a Bayesian network to infer causal and combinatorial relationships among histone modifications and gene expression. A pilot network derived by the same method among polycomb group (PcG) genes and H3K27 trimethylation is accurately supported by current literature. Our unbiased network model among histone modifications is also well supported by cross-validation results. It not only confirmed already known relationships, such as those of H3K27me3 to gene silencing, H3K4me3 to gene activation and the effect of bivalent modification of both H3K4me3 and H3K27me3, but also identified many other relationships that may predict new epigenetic interactions important in epigenetic gene regulation. Our automated inference method, which is potentially applicable to other ChIP-chip or ChIP-seq data analyses, provides a much-needed guide to deciphering the complex histone codes.

PMCID: PMC2493438
PMID: 18562678 [PubMed - indexed for MEDLINE]

(Motifs) (Markov chain Monte Carlo)
17. Nucleic Acids Res. 2008 Jul;36(12):4137-48. Epub 2008 Jun 13.

Extracting sequence features to predict protein-DNA interactions: a comparative study.

Zhou Q, Liu JS.

Department of Statistics, University of California, Los Angeles, CA 90095, USA.
zhou@stat.ucla.edu

Predicting how and where proteins, especially transcription factors (TFs),interact with DNA is an important problem in biology. We present here a systematic study of predictive modeling approaches to the TF-DNA binding problem, which have been frequently shown to be more efficient than those methods only based on position-specific weight matrices (PWMs). In these approaches, a statistical relationship between genomic sequences and gene expression or ChIP-binding intensities is inferred through a regression framework; and influential sequence features are identified by variable selection. We examine a few state-of-the-art learning methods including stepwise linear regression, multivariate adaptive regression splines, neural networks, support vector machines, boosting and Bayesian additive regression trees (BART). These methods are applied to both simulated datasets and two whole-genome ChIP-chip datasets on the TFs Oct4 and Sox2, respectively, in human embryonic stem cells. We find that, with proper learning methods, predictive modeling approaches can significantly improve the predictive power and identify more biologically interesting features, such as TF-TF interactions, than the PWM approach. In particular, BART and boosting show the best and the most robust overall performance among all the methods.

PMCID: PMC2475627
PMID: 18556756 [PubMed - indexed for MEDLINE]

(SNP)
18. BMC Proc. 2007;1 Suppl 1:S56. Epub 2007 Dec 18.

Two-stage approach for identifying single-nucleotide polymorphisms associated with rheumatoid arthritis using random forests and Bayesian networks.

Meng Y, Yang Q, Cuenco KT, Cupples LA, Destefano AL, Lunetta KL.

Genetics Program, Department of Medicine, School of Medicine, Boston University,
715 Albany Street, Boston, Massachusetts 02118, USA.
ymeng@chgr.mgh.harvard.edu

We used the simulated data set from Genetic Analysis Workshop 15 Problem 3 to assess a two-stage approach for identifying single-nucleotide polymorphisms (SNPs) associated with rheumatoid arthritis (RA). In the first stage, we used random forests (RF) to screen large amounts of genetic data using the variable importance measure, which takes into account SNP interaction effects as well as main effects without requiring model specification. We used the simulated 9187 SNPs mimicking a 10 K SNP chip, along with covariates DR (the simulated DRB1 gentoype), smoking, and sex as input to the RF analyses with a training set consisting of 750 unrelated RA cases and 750 controls. We used an iterative RF screening procedure to identify a smaller set of variables for further analysis. In the second stage, we used the software program CaMML for producing Bayesian networks, and developed complex etiologic models for RA risk using the variables identified by our RF screening procedure. We evaluated the performance of this method using independent test data sets for up to 100 replicates.

PMCID: PMC2367609
PMID: 18466556 [PubMed]

(motif) (Markov chain Monte Carlo)
19. PLoS One. 2008 Mar 26;3(3):e1820.

Probabilistic inference of transcription factor binding from multiple data sources.

Lähdesmäki H, Rust AG, Shmulevich I.

Institute for Systems Biology, Seattle, Washington, United States of America.

An important problem in molecular biology is to build a complete understanding of transcriptional regulatory processes in the cell. We have developed a flexible, probabilistic framework to predict TF binding from multiple data sources that differs from the standard hypothesis testing (scanning) methods in several ways. Our probabilistic modeling framework estimates the probability of binding and, thus, naturally reflects our degree of belief in binding. Probabilistic modeling also allows for easy and systematic integration of our binding predictions into other probabilistic modeling methods, such as expression-based gene network inference. The method answers the question of whether the whole analyzed promoter has a binding site, but can also be extended to estimate the binding probability at each nucleotide position. Further, we introduce an extension to model combinatorial regulation by several TFs. Most importantly, the proposed methods can make principled probabilistic inference from multiple evidence sources, such as, multiple statistical models (motifs) of the TFs, evolutionary conservation, regulatory potential, CpG islands, nucleosome positioning, DNase hypersensitive sites, ChIP-chip binding segments and other (prior) sequence-based biological knowledge. We developed both a likelihood and a Bayesian method, where the latter is implemented with a Markov chain Monte Carlo algorithm. Results on a carefully constructed test set from the mouse genome demonstrate that principled data fusion can significantly improve the performance of TF binding prediction methods. We also applied the probabilistic modeling framework to all promoters in the mouse genome and the results indicate a sparse connectivity between transcriptional regulators and their target promoters. To facilitate analysis of other sequences and additional data, we have developed an on-line web tool, ProbTF, which implements our probabilistic TF binding prediction method using multiple data sources. Test data set, a web tool, source codes and supplementary data are available at: http://www.probtf.org.

PMCID: PMC2268002
PMID: 18364997 [PubMed - indexed for MEDLINE]

(Motif) (MCMC)
20. Biostatistics. 2008 Oct;9(4):668-85. Epub 2008 Mar 18.

A transdimensional Bayesian model for pattern recognition in DNA sequences.

Li SM, Wakefield J, Self S.

Division of Oncology Biostatistics, Sidney Kimmel Cancer Center, Johns Hopkins
School of Medicine, Baltimore, MD 21205-2013, USA.

Identification of transcription factor binding sites (TFBSs) is essential to elucidate gene regulatory networks. This article is focused on the recognition of overpresented short patterns, called "motifs", that may correspond to regulatory binding sites in the DNA sequences upstream of genes. An integrated Bayesian model is proposed to incorporate all unknown characteristics in motif discovery, including the number of motifs, motif widths, motif compositions, the number of motif sites, and locations of motif sites. Reversible jump Markov chain Monte Carlo is used to obtain posterior inference in the transdimensional parameter space. We present a number of suggestions for graphical summarization of the posterior distribution over the complex parameter space. The basic model is extended using a third-order Markov structure for nonmotif bases and allowing positions within a motif to be switched between 2 types: "conserved" and "degenerate." We evaluate the prediction accuracy for the simulated data with 3 motifs and apply the model to upstream sequences in high signal-to-noise regions in a human ChIP-chip study. The performance of the Bayesian model is assessed using yeast data sets of various numbers of sequences and background structures, with and without true TFBSs. The performance is also compared to other computational methods, including 2 statistical approaches, AlignACE and multiple expectation maximization for motif elicitation, and 1 word numeration-based approach, yeast motif finder (YMF).

PMID: 18349034 [PubMed - indexed for MEDLINE]

(Motif) (Bayesian Prior, local search and global search; Discriminative motif discovery)

21. BMC Bioinformatics. 2007 Oct 15;8:385.

Discriminative motif discovery in DNA and protein sequences using the DEME algorithm.

Redhead E, Bailey TL.

Institute for Molecular Bioscience, University of Queensland, Brisbane, Qld, 4072
Australia. e.redhead@imb.uq.edu.au

BACKGROUND: Motif discovery aims to detect short, highly conserved patterns in a collection of unaligned DNA or protein sequences. Discriminative motif finding algorithms aim to increase the sensitivity and selectivity of motif discovery by utilizing a second set of sequences, and searching only for patterns that can differentiate the two sets of sequences. Potential applications of discriminative motif discovery include discovering transcription factor binding site motifs in ChIP-chip data and finding protein motifs involved in thermal stability using sets of orthologous proteins from thermophilic and mesophilic organisms. RESULTS: We describe DEME, a discriminative motif discovery algorithm for use with protein and DNA sequences. Input to DEME is two sets of sequences; a "positive" set and a "negative" set. DEME represents motifs using a probabilistic model, and uses a novel combination of global and local search to find the motif that optimally discriminates between the two sets of sequences. DEME is unique among discriminative motif finders in that it uses an informative Bayesian prior on protein motif columns, allowing it to incorporate prior knowledge of residue characteristics. We also introduce four, synthetic, discriminative motif discovery problems that are designed for evaluating discriminative motif finders in various biologically

motivated contexts. We test DEME using these synthetic problems and on two biological problems: finding yeast transcription factor binding motifs in ChIP-chip data, and finding motifs that discriminate between groups of thermophilic and mesophilic orthologous proteins. CONCLUSION: Using artificial data, we show that DEME is more effective than a non-discriminative approach when there are "decoy" motifs or when a variant of the motif is present in the "negative" sequences. With real data, we show that DEME is as good, but not better than non-discriminative algorithms at discovering yeast transcription factor binding motifs. We also show that DEME can find highly informative thermal-stability protein motifs. Binaries for the stand-alone program DEME is free for academic use and is available at http://bioinformatics.org.au/deme/

PMCID: PMC2194741
PMID: 17937785 [PubMed - indexed for MEDLINE]

(Motif) (MCMC)
22. Biometrics. 2008 Jun;64(2):468-78. Epub 2007 Sep 20.

A flexible and powerful bayesian hierarchical model for ChIP-Chip experiments.

Gottardo R, Li W, Johnson WE, Liu XS.

Department of Statistics, University of British Columbia, Vancouver, Canada.
raph@stat.ubc.ca

Chromatin-immunoprecipitation microarrays (ChIP-chip) that enable researchers to identify regions of a given genome that are bound by specific DNA-binding proteins present new challenges for statistical analysis due to the large number of probes, the high noise-to-signal ratio, and the spatial dependence between probes. We propose a method called BAC (Bayesian analysis of ChIP-chip) to detect transcription factor bound regions, which incorporate the dependence between probes while making little assumptions about the bound regions (e.g., length). BAC is robust to probe outliers with an exchangeable prior for the variances, which allows different variances for the probes but still shrink extreme empirical variances. Parameter estimation is carried out using Markov chain Monte Carlo and inference is based on the joint distribution of the parameters. Bound regions are detected using posterior probabilities computed from the joint posterior distribution of neighboring probes. We show that these posterior probabilities are well calibrated and can be used to obtain an estimate of the false discovery rate. The method is illustrated using two publicly available ChIP-chip data sets containing 18 experimentally validated regions. We compare our method to four other baseline and commonly used techniques, namely, the Wilcoxon's rank sum test, TileMap, HGMM, and MAT. We found BAC and HGMM to perform best at detecting validated regions. However, HGMM appears to be very sensitive to probe outliers compared to BAC. In addition, we present a simulation study, which shows that BAC is more powerful than the other four techniques under various simulation scenarios while being robust to model misspecification.

PMID: 17888037 [PubMed - indexed for MEDLINE]

(SNP) (Gibbs sampling)

23. J Dairy Sci. 2007 Oct;90(10):4821-9.

Breeding value estimation for fat percentage using dense markers on Bos Taurus autosome 14.

de Roos AP, Schrooten C, Mullaart E, Calus MP, Veerkamp RF.

HG, 6802 EB Arnhem, The Netherlands. roos.s@hg.nl

Prediction of breeding values using whole-genome dense marker maps for genomic selection has become feasible with the advances in DNA chip technology and the discovery of thousands of single nucleotide polymorphisms in genome-sequencing projects. The objective of this study was to compare the accuracy of predicted breeding values from genomic selection (GS), selection without genetic marker information (BLUP), and gene-assisted selection (GEN) on real dairy cattle data for 1 chromosome. Estimated breeding values of 1,300 bulls for fat percentage, based on daughter performance records, were obtained from the national genetic evaluation and used as phenotypic data. All bulls were genotyped for 32 genetic markers on chromosome 14, of which 1 marker was the causative mutation in a gene with a large effect on fat percentage. In GS, the data were analyzed with a multiple quantitative trait loci (QTL) model with haplotype effects for each marker bracket and a polygenic effect. Identical-by-descent probabilities based on linkage and linkage disequilibrium information were used to model the covariances between haplotypes. A Bayesian method using Gibbs sampling was used to predict the presence of a putative QTL and the effects of the haplotypes in each marker bracket. In BLUP, the haplotype effects were removed from the model, whereas in GEN, the haplotype effects were replaced by the effect of the genotype at the known causative mutation. The breeding values from the national genetic evaluation were treated as true breeding values because of their high accuracy and were used to compute the accuracy of prediction for GS, BLUP, and GEN. The allele substitution effect for the causative mutation, obtained from GEN, was 0.35% fat. The accuracy of the predicted breeding values for GS (0.75) was as high as for GEN (0.75) and higher than for BLUP (0.51). When some markers close to the QTL were omitted from the model, the accuracy of prediction was only slightly lower, around 0.72. The removal of all markers within 8 cM from the QTL reduced the accuracy to 0.64, which was still much higher than BLUP. It is concluded that, when applied to 1 chromosome and if genetic markers close to the QTL are available, the presented model for GS is as accurate as GEN.

PMID: 17881705 [PubMed - indexed for MEDLINE]

(Networks, data integration) (Gibbs sampling)
24. BMC Bioinformatics. 2007 Aug 3;8:283.

Bayesian hierarchical model for transcriptional module discovery by jointly modeling gene expression and ChIP-chip data.

Liu X, Jessen WJ, Sivaganesan S, Aronow BJ, Medvedovic M.

Department of Environmental Health, University of Cincinnati, 3223 Eden Ave, ML
56, Cincinnati, Ohio 45267, USA. xiangdong.liu@cchmc.org

BACKGROUND: Transcriptional modules (TM) consist of groups of co-regulated genes and transcription factors (TF) regulating their

expression. Two high-throughput (HT) experimental technologies, gene expression microarrays and Chromatin Immuno-Precipitation on Chip (ChIP-chip), are capable of producing data informative about expression regulatory mechanism on a genome scale. The optimal approach to joint modeling of data generated by these two complementary biological assays, with the goal of identifying and characterizing TMs, is an important open problem in computational biomedicine. RESULTS: We developed and validated a novel probabilistic model and related computational procedure for identifying TMs by jointly modeling gene expression and ChIP-chip binding data. We demonstrate an improved functional coherence of the TMs produced by the new method when compared to either analyzing expression or ChIP-chip data separately or to alternative approaches for joint analysis. We also demonstrate the ability of the new algorithm to identify novel regulatory relationships not revealed by ChIP-chip data alone. The new computational procedure can be used in more or less the same way as one would use simple hierarchical clustering without performing any special transformation of data prior to the analysis. The R and C-source code for implementing our algorithm is incorporated within the R package gimmR which is freely available at http://eh3.uc.edu/gimm. CONCLUSION: Our results indicate that, whenever available, ChIP-chip and expression data should be analyzed within the unified probabilistic modeling framework, which will likely result in improved clusters of co-regulated genes and improved ability to detect meaningful regulatory relationships. Given the good statistical properties and the ease of use, the new computational procedure offers a worthy new tool for reconstructing transcriptional regulatory networks.

PMCID: PMC1994961
PMID: 17683565 [PubMed - indexed for MEDLINE]

(Classification)

25. Water Environ Res. 2007 Mar;79(3):246-59.

Virulence factor activity relationships: challenges and development approaches.

Tourlousse DM, Stedtfeld RD, Baushke SW, Wick LM, Hashsham SA.

Department of Civil and Environmental Engineering, Michigan State University,
East Lansing 48824, USA.

Virulence factor activity relationships (VFAR) is a predictive approach proposed by the National Research Council's Committee on Drinking Water Contaminants (Washington, D.C.) to classify and rank waterborne pathogens. It is based on the presumption that health threats of waterborne pathogens can be predicted from descriptors at different levels of cellular organization. This paper summarizes challenges that need to be addressed while developing VFAR, with a focus on genomics, such as genomic variability among related pathogens and the need to incorporate genetic descriptors for persistence and host susceptibility. Three key components of VFAR development and validation are also presented, including (1) compilation of a comprehensive VFAR database, (2) development of predictive mathematical models relating descriptors to health effects and other microbial responses, and (3) high-throughput molecular monitoring of drinking water supplies and sources. Bayesian approach

and on-chip polymerase chain reaction are discussed as examples of mathematical models and molecular monitoring.

PMID: 17469656 [PubMed - indexed for MEDLINE]

(Clustering) (Gibbs sampling)
26. Genome Biol. 2007;8(1):R4.

Clustering of genes into regulons using integrated modeling-COGRIM.

Chen G, Jensen ST, Stoeckert CJ Jr.

Department of Bioengineering, University of Pennsylvania, Philadelphia,
Pennsylvania 19104, USA. ggchen@pcbi.upenn.edu

We present a Bayesian hierarchical model and Gibbs Sampling implementation that integrates gene expression, ChIP binding, and transcription factor motif data in a principled and robust fashion. COGRIM was applied to both unicellular and mammalian organisms under different scenarios of available data. In these applications, we demonstrate the ability to predict gene-transcription factor interactions with reduced numbers of false-positive findings and to make predictions beyond what is obtained when single types of data are considered.

PMCID: PMC1839128
PMID: 17204163 [PubMed - indexed for MEDLINE]

(Microscopy)
27. Ultrasonics. 2006 Dec;45(1-4):82-91. Epub 2006 Jul 31.
Effect of sparse basis selection on ultrasonic signal representation.

Zhang GM, Harvey DM, Braden DR.

General Engineering Research Institute, Liverpool John Moores University,
Liverpool, United Kingdom. g.zhang@ljmu.ac.uk <g.zhang@ljmu.ac.uk>

Recently, adaptive sparse representations of ultrasonic signals have been utilized to improve the performance of scanning acoustic microscopy (SAM), a common nondestructive tool for failure analysis of microelectronic packages. The adaptive sparse representation of an ultrasonic signal is generated by decomposing it in a learned overcomplete dictionary using a sparse basis selection algorithm. Detection and location of ultrasonic echoes are then performed on the basis of the resulting redundant representation. This paper investigates the effect of sparse basis selection algorithms on ultrasonic signal representation. The overcomplete independent component analysis, focal underdetermined system solver (FOCUSS), and sparse Bayesian learning algorithms are examined. Numerical simulations are performed to quantitatively analyze the efficiency of ultrasonic signal representations. Experiments with ultrasonic A-scans acquired from flip-chip packages are also carried out in the study. The efficiency of ultrasonic signal representations are evaluated in terms of the different criteria that can be used to measure its performance for different SAM applications, such as waveform estimation, echo detection, echo location and C-scan imaging. The results show that the FOCUSS algorithm performs best overall.

PMID: 16930664 [PubMed - indexed for MEDLINE]

(Motif) (Gibbs sampling)
28. PLoS Comput Biol. 2005 Dec;1(7):e67. Epub 2005 Dec 9.
PhyloGibbs: a Gibbs sampling motif finder that incorporates phylogeny.
Siddharthan R, Siggia ED, van Nimwegen E.
Center for Studies in Physics and Biology, The Rockefeller University, New York,
New York, United States of America.

A central problem in the bioinformatics of gene regulation is to find the binding sites for regulatory proteins. One of the most promising approaches toward identifying these short and fuzzy sequence patterns is the comparative analysis of orthologous intergenic regions of related species. This analysis is complicated by various factors. First, one needs to take the phylogenetic relationship between the species into account in order to distinguish conservation that is due to the occurrence of functional sites from spurious conservation that is due to evolutionary proximity. Second, one has to deal with the complexities of multiple alignments of orthologous intergenic regions, and one has to consider the possibility that functional sites may occur outside of conserved segments. Here we present a new motif sampling algorithm, PhyloGibbs, that runs on arbitrary collections of multiple local sequence alignments of orthologous sequences. The algorithm searches over all ways in which an arbitrary number of binding sites for an arbitrary number of transcription factors (TFs) can be assigned to the multiple sequence alignments. These binding site configurations are scored by a Bayesian probabilistic model that treats aligned sequences by a model for the evolution of binding sites and "background" intergenic DNA. This model takes the phylogenetic relationship between the species in the alignment explicitly into account. The algorithm uses simulated annealing and Monte Carlo Markov-chain sampling to rigorously assign posterior probabilities to all the binding sites that it reports. In tests on synthetic data and real data from five Saccharomyces species our algorithm performs significantly better than four other motif-finding algorithms, including algorithms that also take phylogeny into account. Our results also show that, in contrast to the other algorithms, PhyloGibbs can make realistic estimates of the reliability of its predictions. Our tests suggest that, running on the five-species multiple alignment of a single gene's upstream region, PhyloGibbs on average recovers over 50% of all binding sites in S. cerevisiae at a specificity of about 50%, and 33% of all binding sites at a specificity of about 85%. We also tested PhyloGibbs on collections of multiple alignments of intergenic regions that were recently annotated, based on ChIP-on-chip data, to contain binding sites for the same TF. We compared PhyloGibbs's results with the previous analysis of these data using six other motif-finding algorithms. For 16 of 21 TFs for which all other motif-finding methods failed to find a significant motif, PhyloGibbs did recover a motif that matches the literature consensus. In 11 cases where there was disagreement in the results we compiled lists of known target genes from the literature, and found that running PhyloGibbs on their regulatory regions yielded a binding motif matching the literature consensus in all but one of the cases. Interestingly, these literature gene lists had little overlap with the targets annotated based on the ChIP-on-chip data. The PhyloGibbs code can be downloaded from

http://www.biozentrum.unibas.ch/~nimwegen/cgi-bin/phylogibbs.cgi or http://www.imsc.res.in/~rsidd/phylogibbs. The full set of predicted sites from our tests on yeast are available at http://www.swissregulon.unibas.ch.

PMCID: PMC1309704
PMID: 16477324 [PubMed - indexed for MEDLINE]

(Data integration)

29. Genome Inform. 2005;16(1):83-94.
Integrating genomic data to predict transcription factor binding.
Holloway DT, Kon M, DeLisi C.
Molecular Biology Cell Biology and Biochemistry, Boston University, Boston, MA
02215, USA. dth128@bu.edu

Transcription factor binding sites (TFBS) in gene promoter regions are often predicted by using position specific scoring matrices (PSSMs), which summarize sequence patterns of experimentally determined TF binding sites. Although PSSMs are more reliable than simple consensus string matching in predicting a true binding site, they generally result in high numbers of false positive hits. This study attempts to reduce the number of false positive matches and generate new predictions by integrating various types of genomic data by two methods: a Bayesian allocation procedure, and support vector machine classification. Several methods will be explored to strengthen the prediction of a true TFBS in the Saccharomyces cerevisiae genome: binding site degeneracy, binding site conservation, phylogenetic profiling, TF binding site clustering, gene expression profiles, GO functional annotation, and k-mer counts in promoter regions. Binding site degeneracy (or redundancy) refers to the number of times a particular transcription factor's binding motif is discovered in the upstream region of a gene. Phylogenetic conservation takes into account the number of orthologous upstream regions in other genomes that contain a particular binding site. Phylogenetic profiling refers to the presence or absence of a gene across a large set of genomes. Binding site clusters are statistically significant clusters of TF binding sites detected by the algorithm ClusterBuster. Gene expression takes into account the idea that when the gene expression profiles of a transcription factor and a potential target gene are correlated, then it is more likely that the gene is a genuine target. Also, genes with highly correlated expression profiles are often regulated by the same TF(s). The GO annotation data takes advantage of the idea that common transcription targets often have related function. Finally, the distribution of the counts of all k-mers of length 4, 5, and 6 in gene's promoter region were examined as means to predict TF binding. In
each case the data are compared to known true positives taken from ChIP-chip data, Transfac, and the Saccharomyces Genome Database. First, degeneracy, conservation, expression, and binding site clusters were examined independently and in combination via Bayesian allocation. Then, binding sites were predicted with a support vector machine (SVM) using all methods alone and in combination. The SVM works best when all genomic data are combined, but can also identify which methods contribute the most to accurate classification. On average, a support vector machine can classify binding sites with high sensitivity and an accuracy of almost 80%.

PMID: 16362910 [PubMed - indexed for MEDLINE]

(Data noise)

30. J Comput Biol. 2003;10(3-4):433-52.
Bayesian estimation of transcript levels using a general model of array measurement noise.
Dror RO, Murnick JG, Rinaldi NJ, Marinescu VD, Rifkin RM, Young RA.

Department of Electrical Engineering and Computer Science, Massachusetts Institute of Technology, Cambridge, MA 02139, USA. rondror@ai.mit.edu

Gene arrays demonstrate a promising ability to characterize expression levels across the entire genome but suffer from significant levels of measurement noise. We present a rigorous new approach to estimate transcript levels and ratios from one or more gene array experiments, given a model of measurement noise and available prior information. The Bayesian estimation of array measurements (BEAM) technique provides a principled method to identify changes in expression level, combine repeated measurements, or deal with negative expression level measurements. BEAM is more flexible than existing techniques, because it does not assume a specific functional form for noise and prior models. Instead, it relies on computational techniques that apply to a broad range of models. We use Affymetrix yeast chip data to illustrate the process of developing accurate noise and prior models from existing experimental data. The resulting noise model includes novel features such as heavy-tailed additive noise and a gene-specific bias term. We also verify that the resulting noise and prior models fit data from an Affymetrix human chip set.

PMID: 12935337 [PubMed - indexed for MEDLINE]

(Review paper)
31. Curr Opin Drug Discov Devel. 2002 May;5(3):428-38.
Multiscale and Bayesian approaches to data analysis in genomics high-throughput screening.

Yang C, Bakshi BR, Rathman JF, Blower PE Jr.

LeadScope Inc, 1245 Kinnear Road, Columbus, OH 43212, USA. cyang@leadscope.com

Tremendous amounts of data are produced by high-throughput screening methods currently employed in drug discovery and product development. A typical cDNA microarray or oligonucleotide-based gene chip experiment easily generates over 10,000 data points for each array or chip. The challenge of inferring meaningful information is formidable given the size and number of these datasets. This paper reviews the current status of statistical tools available for gene expression analysis, with emphasis on Bayesian approaches and multiscale wavelet filtering. Fundamental concepts of Bayesian and multiscale modeling are discussed from the perspective of their potential to address important issues related to the analysis of gene expression data, such as the fact that genomic data often have non-Gaussian distributions and feature localization and multiple scales in both frequency and measurement dimension. Recent publications in these areas are reviewed. Wavelet filtering and the advantages of multiscale methods are demonstrated by application to publicly available gene expression data from the National Cancer Institute (NCI). Multiscale methods, including multiscale principal component analysis (MSPCA), are applied to extract gene subsets and to

visualize data in multidimensions for comparisons. Similarity in cell lines and gene selection are effectively visualized and quantitatively compared.
PMID: 12058619 [PubMed - indexed for MEDLINE]

(Microarray gene expression data analysis)
32. Bioinformatics. 2002 Apr;18(4):566-75.
Application of Bayesian decomposition for analysing microarray data.

Moloshok TD, Klevecz RR, Grant JD, Manion FJ, Speier WF 4th, Ochs MF.

Bioinformatics Working Group, Fox Chase Cancer Center, Philadelphia, PA 19111,
USA. td_moloshok@fccc.edu

MOTIVATION: Microarray and gene chip technology provide high throughput tools for measuring gene expression levels in a variety of circumstances, including cellular response to drug treatment, cellular growth and development, tumorigenesis, among many other processes. In order to interpret the large data sets generated in experiments, data analysis techniques that consider biological knowledge during analysis will be extremely useful. We present here results showing the application of such a tool to expression data from yeast cell cycle experiments. RESULTS: Originally developed for spectroscopic analysis, Bayesian Decomposition (BD) includes two features which make it useful for microarray data analysis: the ability to assign genes to multiple coexpression groups and the ability to encode biological knowledge into the system. Here we demonstrate the ability of the algorithm to provide insight into the yeast cell cycle, including identification of five temporal patterns tied to cell cycle phases as well as the identification of a pattern tied to an approximately 40 min cell cycle oscillator. The genes are simultaneously assigned to the patterns, including partial assignment to multiple patterns when this is required to explain the expression profile. AVAILABILITY: The application is available free to academic users under a material transfer agreement. Go to http://bioinformatics.fccc.edu/ for more details.

PMID: 12016054 [PubMed - indexed for MEDLINE]

(Medicine)
33. Clin Chem. 1998 Feb;44(2):420-7.

Development of new cancer chemoprevention agents: role of pharmacokinetic/pharmacodynamic and intermediate endpoint biomarker monitoring.

Lieberman R, Crowell JA, Hawk ET, Boone CW, Sigman CC, Kelloff GJ.

Chemoprevention Branch, National Cancer Institute, Rockville, MD 20852, USA.
Liebermr@dcpcepn.nci.nih.gov

Recently, several promising strategies have been advanced for improving the efficiency of new agent development. These include pharmacokinetic/pharmacodynamic (PK/PD) and intermediate endpoint biomarker (IEB) monitoring. Here, we review their essential role as practical tools for guiding the evaluation of agents for cancer chemoprevention (CP) and provide examples of CP agents that utilize these approaches. Several important categories of IEBs are

delineated, including histologically based (intraepithelial neoplasias and nuclear morphometry). The use of select IEBs combined with a Bayesian method for clinical trial monitoring for rapid identification of ineffective or promising agents is discussed. The similarities between IEB and TDM are described. Finally, we present future tools for enhanced monitoring of CP agents that will impact on laboratory medicine and are also applicable to many other drug classes, e.g., laser capture microdissection and cDNA chip microarrays that assess geneexpression patterns of precancerous and cancerous lesions.

PMID: 9474054 [PubMed - indexed for MEDLINE]

Supplementary References

1. Pearlmutter BA: **Fast exact multiplication by the Hessian**. *Neural Computation* 1994, **6**(1).
